# Supplementary material for: Combining sound with tongue stimulation for the treatment of tinnitus: a multi-site single-arm controlled pivotal trial
Source: Nat Commun. 2024 Aug 19;15:6806. doi: 10.1038/s41467-024-50473-z (PMC11333749; doi:10.1038/s41467-024-50473-z)
Supplement: Supplementary file 1 — Supplementary Information [file 41467_2024_50473_MOESM1_ESM.pdf]

Supplementary Information

Combining sound with tongue stimulation for the treatment of tinnitus: a controlled pivotal trial

Supplementary Fig. 1: Distribution of the Tinnitus Handicap Inventory (THI) scores across participants at screening visit.

Supplementary Table 1: Total participants not enrolled for those who were screened in clinic.

Supplementary Table 2: Total participants who did not meet inclusion criteria for those who were screened in clinic.

Supplementary Table 3: Total participants who met exclusion criteria for those who were screened in clinic.

Supplementary Fig. 2: Treatment compliance of participants in TENT-A3 study.

Supplementary Fig. 3: Hearing thresholds for enrolled participants.

Supplementary Table 4: Primary endpoint analyses by sex (male) based on Tinnitus Handicap Inventory (THI).

Supplementary Table 5: Primary endpoint analyses by sex (female) based on Tinnitus Handicap Inventory (THI).

Supplementary Table 6: Additional endpoint analyses based on Tinnitus Functional Index (TFI).

Real-world evidence (RWE) data: summary of methods and results.

Supplementary Table 7: Demographics of all available real-world evidence (RWE) data n=276) and patients who attended both initial and 6-week assessments (n=204).

Supplementary Fig. 4: Real-world evidence (RWE) violin plots for symptoms of tinnitus based on Tinnitus Handicap Inventory (THI) for the different severity groups from initial assessment to 6-week assessment (n=204).

Supplementary Table 8: Conversion of non-responders to responders with bimodal treatment for Intention-to-Treat (ITT) population-full cohort (n=112).

Supplementary Table 9: Additional endpoint analyses based on Health Utilities Index 3 (HUI3).

TENT-A3 Clinical Investigation Plan

TENT-A3 Statistical Analysis Plan

Supplementary Fig. 1: Distribution of the Tinnitus Handicap Inventory (THI) scores across participants at screening visit.

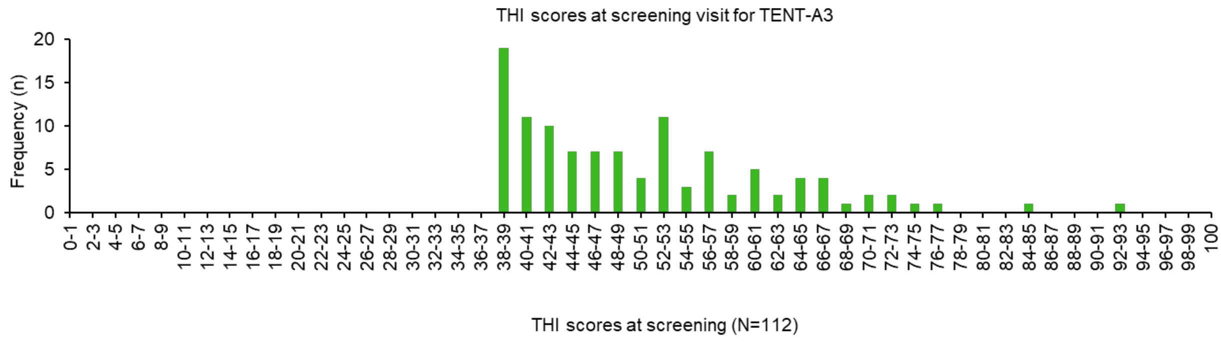

Supplementary Table 1: Total participants not enrolled for those who were screened in clinic.

**Participants not enrolled in study**	**Percentage, % (number of participants/total)**
**Eligible but not enrolled**	n=15
1. Patient withdrew prior to enrolment	26.7% (4/15)
2. Patients on waitlist (not enrolled)	73.3% (11/15)

Supplementary Table 2: Total participants who did not meet inclusion criteria for those who were screened in clinic.

Participants not enrolled in study	Percentage, % (number of participants/total)
**Screen failures. Participants did not meet inclusion criteria**	n=95
1. 18 years and over at time of consent	0.0% (0/95)
2. Ability to read and understand Dutch/Flemish/English or German (depending on clinical site)	0.0% (0/95)
3. Willing and able to provide and understand informed consent	0.0% (0/95)
4. Willing to commit to the full duration of the investigation	1.1% (1/95)
5. Subjective tinnitus	1.1% (1/95)
6. Tinnitus duration for greater than or equal to 3 months and less than or equal to 10 years at time of consent	4.2% (4/95)
7. Baseline Tinnitus Handicap Inventory (THI) greater than or equal to 38	34.7% (33/95)

Patients who did not meet inclusion criteria include those with missing responses to inclusion criteria.

Supplementary Table 3: Total participants who met exclusion criteria for those who were screened in clinic.

Participants not enrolled in study	Percentage, % (number of participants/total)
**Screen failures. Participants met exclusion criteria**	n=95
1. Subjective tinnitus where pulsatility is the dominant feature (participant reported)	8.4% (8/95)
2. Objective tinnitus, where the tinnitus is also observed by the examiner	0.0% (0/95)
3. Commenced usage of hearing aid within the last 90 days	2.1% (2/95)
4. Meniere's disease	0.0% (0/95)
5. Hospitalization, or visit to a physician, for a head or neck injury, including whiplash, in the previous 12 months	1.1% (1/95)
6. Temporomandibular Joint Disorder (TMJ)	1.1% (1/95)
7. Pregnancy	0.0% (0/95)
8. Oral piercings that cannot or will not be removed for the second stage of the investigation	0.0% (0/95)
9. Neurological condition that may lead to seizures or loss of consciousness (e.g. epilepsy)	0.0% (0/95)
10. Severe cognitive impairment based on Mini-Mental State Examination (MMSE, less than 20)	0.0% (0/95)
11. Patient with a pacemaker or other electro-active implanted device	0.0% (0/95)
12. Abnormal findings following otoscopy/tympanometry that may be contributing to or causing the tinnitus as assessed by an Audiologist/ENT	8.4% (8/95)
13. Initiated new prescription medications or medical treatments in the previous 3 months that may impact the outcomes of the investigation, by discretion of the investigator	14.7% (14/95)
14. Ceased prescription medications or medical treatments in the previous 3 months that may impact the outcomes of the investigation, by discretion of the investigator	2.1% (2/95)
15. State-Trait Anxiety Inventory (STAI) score of >120	0.0% (0/95)
16. Current or previous involvement in medico-legal cases (self-reported)	0.0% (0/95)
17. Participant previously diagnosed with psychosis or schizophrenia	0.0% (0/95)
18. Participants diagnosed with Burning Mouth Syndrome (BMS)	0.0% (0/95)
19. Previous use of Lenire®	0.0% (0/95)
20. Previous involvement in a clinical investigation for tinnitus or had an experimental/surgical treatment for tinnitus	20.0% (19/95)
21. Hearing loss of greater than 80dB HL in any test frequency in the set {2k,3k,4k,6k,8k} Hz or greater than 40 dB HL in the set {250,500,1k} Hz either unilaterally or bilaterally	22.1% (21/95)
22. The site Principal Investigator (PI) does not deem the candidate to be suitable for the investigation for other reasons not listed above. Rationale must be provided.	13.7% (13/95)

Patients who met exclusion criteria include those with missing responses to exclusion criteria.
dB HL: decibels in hearing level.

Supplementary Fig. 2: Treatment compliance of participants in TENT-A3 study.

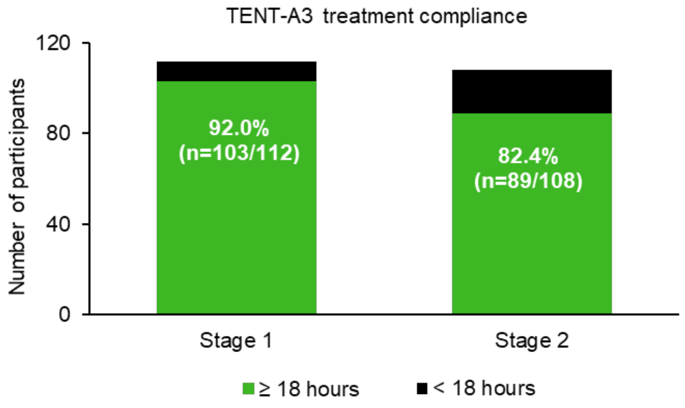

Number of participants who were compliant (at least 18 hours of device usage) for each stage of treatment. There were missing data for four participants in Stage 2. Two of these participants were compliant in Stage 1 but were withdrawn before starting Stage 2 (one participant was withdrawn by investigators due to starting a new prescription medication as per exclusion criteria and one participant was withdrawn by investigators due to aggressive behavior and non-commitment to treatment). The two other participants were non-compliant in Stage 1 and did not continue with Stage 2 of the study (one participant had an AE that had been determined to be not device related and did not want to continue with Stage 2 and one participant withdrew less than 2 weeks after starting Stage 1 as they did not want to continue in the study).

Supplementary Fig. 3: Hearing thresholds for enrolled participants.

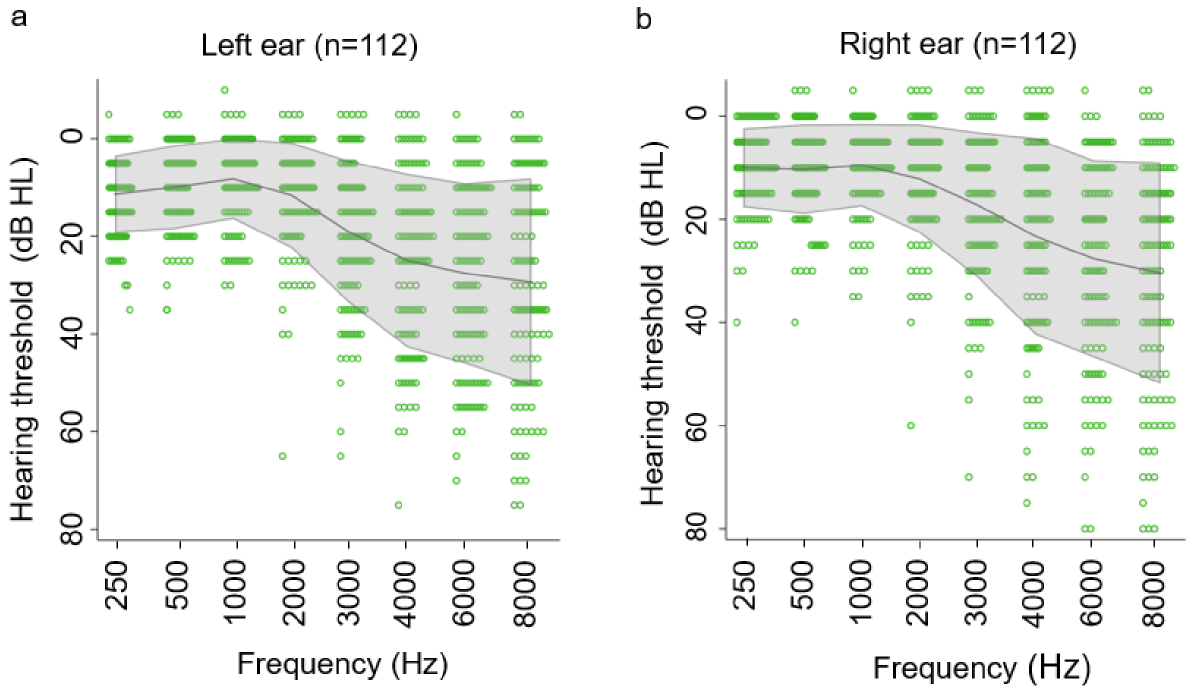

Data are plotted for each participant and presented for left (**a**) or right ear (**b**). Circle represents threshold value for each frequency and participant, solid line corresponds to mean threshold value for each frequency across participants, and shaded region corresponds to standard deviation of threshold values for each frequency across participants. Data points are jittered for visibility. dB HL: decibels in hearing level.

Supplementary Table 4: Primary endpoint analyses by sex (male) based on Tinnitus Handicap Inventory (THI).

ITT population - full **male** cohort (n=77)			
	**Sound therapy (Stage 1)**	**Addition of tongue stimulation to sound therapy (Stage 2)**	**Full 12-weeks of treatment (Stages 1 and 2)**
Estimate ± SE	64.9% ± 5.4%	40.3% ± 5.6%	77.9% ± 4.7%
95% CI	53.8%, 74.7%	30.0%, 51.4%	67.5%, 85.7%
ITT population - moderate or worse severity group (THI ≥ 38, n=24)			
	**Sound therapy (Stage 1)**	**Addition of tongue stimulation to sound therapy (Stage 2)**	**Full 12-weeks of treatment (Stages 1 and 2)**
Estimate ± SE	45.8% ± 10.2%	58.3% ± 10.1%	75.0% ± 8.8%
95% CI	27.9%, 64.9%	38.8%, 75.5%	55.1%, 88.0%

ITT: Intention-to-Treat

Supplementary Table 5: Primary endpoint analyses by sex (female) based on Tinnitus Handicap Inventory (THI).

	ITT population - full **female** cohort (n=35)		
	**Sound therapy (Stage 1)**	**Addition of tongue stimulation to sound therapy (Stage 2)**	**Full 12-weeks of treatment (Stages 1 and 2)**
Estimate ± SE	60.0% ± 8.3%	51.4% ± 8.4%	85.7% ± 5.9%
95% CI	43.6%, 74.4%	35.6%, 67.0%	70.6%, 93.7%
	ITT population - moderate or worse severity group (THI ≥ 38, n=20)		
	**Sound therapy (Stage 1)**	**Addition of tongue stimulation to sound therapy (Stage 2)**	**Full 12-weeks of treatment (Stages 1 and 2)**
Estimate ± SE	40.0% ± 11.0%	60.0% ± 11.0%	80.0% ± 8.9%
95% CI	21.9%, 61.3%	38.7%, 78.1%	58.4%, 91.9%

ITT: Intention-to-Treat

Supplementary Table 6: Additional endpoint analyses based on Tinnitus Functional Index (TFI).

	ITT population - all severity groups (n=112)		
	**Sound therapy (Stage 1)**	**Addition of tongue stimulation to sound therapy (Stage 2)**	**Full 12-weeks of treatment (Stages 1 and 2)**
Estimate ± SE	41.1% ± 4.6%	33.9% ± 4.5%	51.8% ± 4.7%
95% CI	32.4, 50.3%	25.8%, 43.1%	42.6%, 60.8%
	ITT population - moderate or worse severity group (THI ≥ 38, n=44)		
	**Sound therapy (Stage 1)**	**Addition of tongue stimulation to sound therapy (Stage 2)**	**Full 12-weeks of treatment (Stages 1 and 2)**
Estimate ± SE	29.6% ± 6.9%	45.5% ± 7.5%	50.0% ± 7.5%
95% CI	18.2%, 44.2%	31.7%, 59.9%	38.8%, 64.2%

A responder is defined as a participant with an improvement in Tinnitus Functional Index (TFI) score of at least 13 points. ITT: Intention-to-Treat.

Real-world evidence (RWE) data: summary of methods and results

The purpose of the Retrospective Chart Review was to report on the performance of the Lenire device using real-world evidence (RWE) collected in the Otologie Clinic in Dublin, Ireland. The study was a single site, single-arm observational retrospective review of patients who attended Otologie for treatment with the Lenire device from March 2021 to June 2022. There was a total of 276 patients who presented at the Otologie Clinic with tinnitus symptoms, were eligible for Lenire, and consented to the Lenire standard of care procedure which included an initial assessment, a device fitting visit, and a follow-up assessment at 6-weeks from commencement of treatment.

To be eligible for Lenire, patients must present with chronic subjective tinnitus (> 3 months), be 18 years of age or older, and have a hearing profile of ≤ 40 dB HL in the frequency range of 250 Hz – 1000 Hz and ≤ 80 dB HL in the frequency range of 2000 Hz-8000 Hz. Contraindications included those who have a pacemaker, defibrillator, or any other active implantable device; are pregnant; have epilepsy or other conditions which may cause loss of consciousness; have conditions that cause impaired sensitivity of the tongue; or have lesions, sore or inflammation of the oral cavity.

Primary outcome measure was the mean changes in severity of tinnitus (measured using the Tinnitus Handicap Inventory (THI)) from treatment initiation of bimodal treatment to the 6-week assessment in different tinnitus severity groups. Ethical approval from the Research Ethics Committee of the Tallaght University Hospital-St James's Hospital (Project ID: 0517) was obtained to use the data of patients collected by the Otologie Clinic starting from March 2021, which is when the clinic started using a Customer Relationship Management (CRM) system for collecting relevant patient data.

The database was verified against the source data (i.e., HubSpot) by means of an on-site Monitoring Visit carried out by an external Clinical Research Organization (CRO: Avania, Bilthoven, Netherlands). Source Data Verification (SDV) was performed on all variables for 20% of the data of all subjects included in the export of this Retrospective Chart Review. No discrepancies were found during the SDV. Monitoring was performed by a qualified and trained Clinical Research Associate from Avania. The selected monitor was independent from the investigation site.

A total of 276 patients attended Otologie Clinic for treatment with the Lenire device from March 1, 2021 to June 30, 2022. Of the 276 patients, 96.7% completed the initial assessment, and 75.4% completed the 6-week assessment. There were 204 patients who completed the THI at both visits to enable THI analysis for this RWE data. **Supplementary Table 7** provides an overview of the demographics of the patients collected at the initial assessment (276 patients) and also the same demographics data but for the 204 patients who had THI scores for both the initial assessment and 6-weeks assessment. Comparison of characteristics and values shows that the demographics were similar for both groups, i.e., the patients not included in the THI analysis did not have major differences in demographics compared to those included in the RWE THI analysis, minimizing bias attributed to missing data.

When performing stratified analysis of the data based on THI severity categories (i.e., none/slight, mild, moderate, severe, and catastrophic groups), there was a larger mean reduction in tinnitus symptoms for more severely bothered patients from initial assessment to 6-week assessment (mean change in THI score of 8.5, -4.4, -9.2, -15.7, and -26.1 points, respectively; **Supplementary Fig. 4a**). The patients were separately divided into two broader severity groups, one with those who are sufficiently bothered by their tinnitus (THI $\geq$ 38 including moderate, severe, and catastrophic groups) and those who are either minimally or not bothered by their tinnitus (THI < 38). There was a THI improvement of -14.5 points (95% CI:

-17.6, -11.4) for the THI ≥ 38 group and -2.8 points (95% CI: -5.8, 0.3) for the THI < 38 group (**Supplementary Fig. 4b**). Applying an independent t-test on the mean improvements in THI score between the two groups results in a p-value less than 0.001.

Field Product Experience Reports (FPERs) were collected as part of the vigilance reporting requirements for medical devices available commercially in the EU as per the EU Medical Device Regulations. There were no field safety reports that required reporting to the Competent Authorities; neither has any Field Safety Corrective Action, Field Safety Notice, or Product Recall been required. The fact that none of these actions have been required highlights the low risk associated with the device in the real-world situation.

In summary, the clinical efficacy and safety reports established from the review of the real-world clinical data support a high benefit to risk profile for the Lenire treatment in improving tinnitus symptoms. In particular, these clinical data from a real-world situation reveal that greater performance or efficacy of Lenire bimodal stimulation treatment is observed in patients with a higher symptom severity, in which patients sufficiently bothered by their tinnitus (THI ≥ 38) when starting bimodal treatment achieved significantly larger improvements in tinnitus symptoms compared to those with less or not bothersome tinnitus (THI < 38).

Supplementary Table 7: Demographics of all available real-world evidence (RWE) data N=276) and patients who attended both initial and 6-week assessments (n=204).

**All available patient data (n=276)**	
**Age (years)**	
Mean ± SD (n)	51.1 ± 12.8 (276)
**Sex [% (n/N)]**	
Male	80.7% (222/275)
Female	19.3% (53/275)
**Tinnitus duration (months or years) [% (n/N)]**	
< 6 months	11.3% (31/275)
6 months to < 5 years	48.0% (132/275)
5 years to < 10 years	16.4% (45/275)
10 years to < 20 years	12.7% (35/275)
20 years and more	9.1% (25/275)
Not sure	2.5% (7/275)
**Cohort of patients who attended initial assessment and 6-week assessment (n=204)**	
**Age (years)**	
Mean ± SD (n)	51.2 ± 12.9 (204)
**Sex [% (n/N)]**	
Male	80.8% (164/203)
Female	19.2% (39/203)
**Tinnitus duration (months or years) [% (n/N)]**	
< 6 months	12.8% (26/204)
6 months to < 5 years	48.5% (99/204)
5 years to < 10 years	14.7% (30/204)
10 years to < 20 years	13.7% (28/204)
20 years and more	8.3% (17/204)
Not sure	2.0% (4/204)

Comparison of these two cohorts shows that the demographics were similar for both groups; there were no major differences in demographics among those who were not included in the Tinnitus Handicap Inventory (THI) analysis compared to those who were included in the analysis shown in **Supplementary Fig. 4**. Note that answers were missing for several items across two patients, where n values do not always sum to 276 (top half) or 204 (bottom half).

Supplementary Fig. 4: Real-world evidence (RWE) violin plots for symptoms of tinnitus based on Tinnitus Handicap Inventory (THI) for the different severity groups from initial assessment to 6-week assessment (n=204).

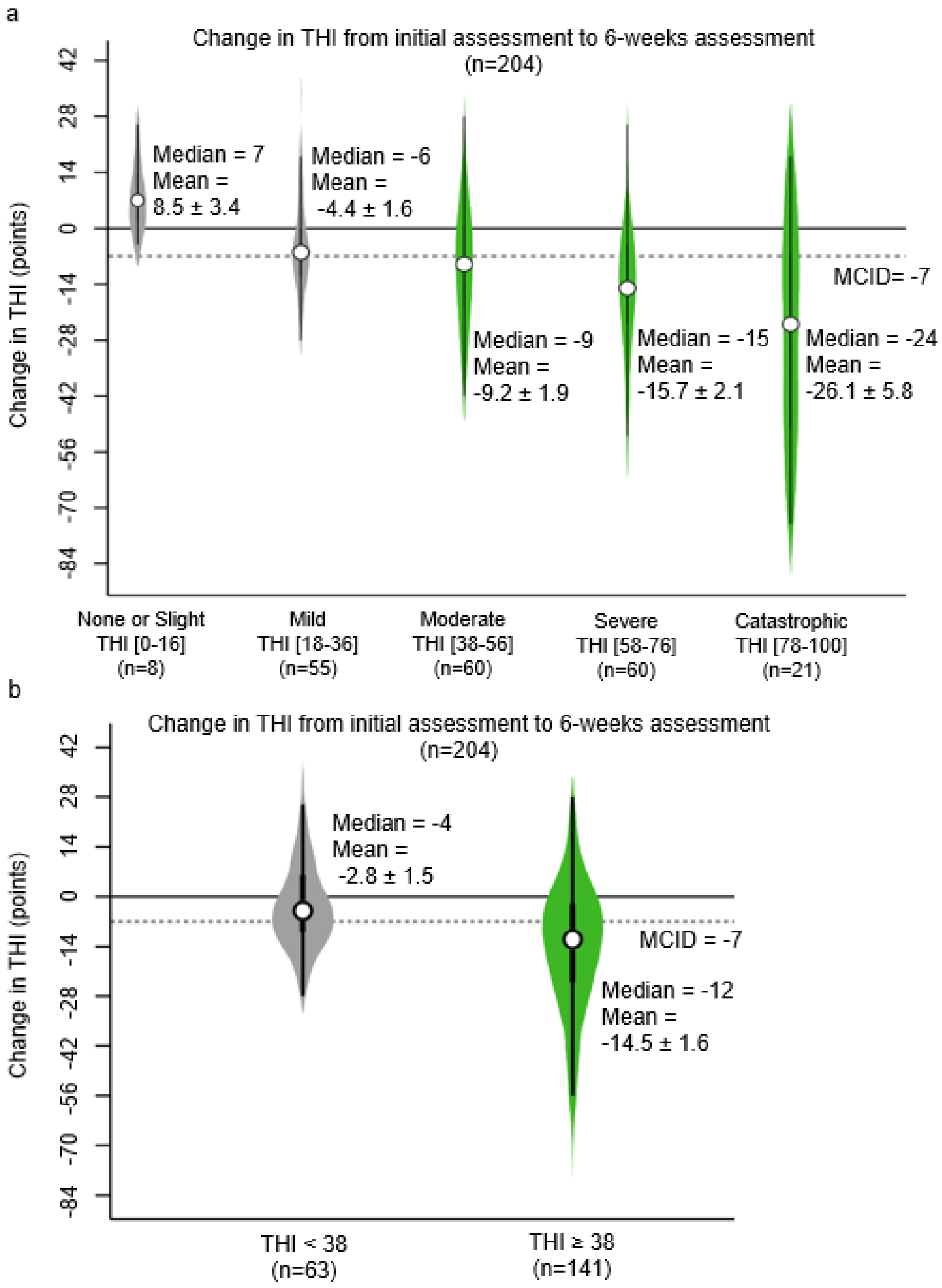

(**a**) The severity groups included five standard Tinnitus Handicap Inventory (THI) categories: none/slight (0-16), mild (18-36), moderate (38-56), severe (58-76), and catastrophic (78-100). (**b**) Two broader clinically relevant severity groups were also included: THI < 38 group (n=63) and THI ≥ 38 group (n=141). There was a significant difference between the two clinically relevant THI severity groups (independent t-test for comparison between groups: p-value < 0.001). MCID: Minimal clinically important difference.

Supplementary Table 8: Conversion of non-responders to responders with bimodal treatment for Intention-to-Treat (ITT) population-full cohort (n=112).

Conversion of non-responders to responders with bimodal treatment<br>ITT population - full cohort (n=112)		
	**Non-responder to sound therapy**	**Non-responder to sound therapy but responded with addition of tongue stimulation to sound therapy**
Estimate ± SE	36.7% ± 4.6%	64.9% ± 7.6%
95% CI	27.8%, 45.7%	50.0%, 79.7%

A responder to bimodal treatment in Stage 2 is defined as a participant with an improvement in Tinnitus Handicap Inventory (THI) score of at least 7 points above what was already achieved during Stage 1 with sound-only stimulation. The responder rate ± SE and corresponding 95% CI of the different groups in the ITT population are presented in the table. The generation of the 95% CIs are described in the Materials and Methods section.

Supplementary Table 9: Additional endpoint analyses based on Health Utilities Index 3 (HUI3).

Mean HUI3 total score - Complete cases (n=80)			
	**Screening visit**	**Interim visit**	**Final visit**
Mean ± SD	0.8 ± 0.19	0.84 ± 0.18	0.85 ± 0.19
95% CI	0.77, 0.86	0.80, 0.88	0.80, 0.89

The HUI3 total score ranges from 0.0 (considered dead) to 1.0 (healthy).

Treatment Evaluation of Neuromodulation for Tinnitus – Stage A3

TENT-A3

Clinical Investigation Plan Number: CN0072
National Clinical Trial (NCT) Identified Number: NCT05227365

[redacted]

Sponsor and Manufacturer
Neuromod Devices Limited
[redacted]
R2YP, Ireland

CONFIDENTIALITY STATEMENT
This document contains confidential information that must not be disclosed to anyone other than the Sponsor, the Investigator Team, Clinical Research Organisation (CRO), Regulatory Authorities or members of the Ethics Committees (ECs), unless authorised to do so.

INVESTIGATION REFERENCE NUMBERS

**Sponsor**	Neuromod Devices Limited<br>[redacted]<br>Ireland
**Funder**	Neuromod Devices Limited
**Ethics Committee Numbers**	A list of Ethics Committee Numbers will be maintained by the Sponsor/CRO.
**Sponsor Reference Number**	CN0072
**Revision Number and Date**	Revision v5.0<br>See DCR22247

[redacted]

CONFIDENTIAL: May not be reproduced, distributed or disclosed without prior consent from Neuromod Device Limited

[redacted]

**Designated Clinical Research Organisation**	Avania B.V,<br>[redacted]<br>3723 MB Bilthoven<br>The Netherlands<br>[redacted]

CONFIDENTIAL: May not be reproduced, distributed or disclosed without prior consent from Neuromod Device Limited

1.0 Summary

**Investigation Title**	Treatment Evaluation of Neuromodulation for Tinnitus – Stage A3
**Clinical Investigation Plan Number**	CN0072
**Investigation Phase**	Post-Market Clinical Follow-Up
**Investigation Design**	Multi-site, single-arm repeated measures prospective investigation
**Investigation Population**	Adults (at least 18 years of age) with subjective tinnitus, with a minimum tinnitus severity score and duration.
**Investigators and Investigations Sites**	A list of investigational site(s), principal investigator(s) and corresponding coordinating investigators is kept separately from this clinical investigation plan. The study is performed in a clinical environment that is representative of the intended normal conditions of the use of the *Lenire*® device and the target subject population.
**Planned Number of Participants**	Up to 112 participants (including up to 20% allowance for drop-outs). 89 participants are intended to complete all assessments.
**Follow-Up Duration**	12 weeks post enrolment
**Planned Visits**	• Visit 1: SCREENING (Week -10 (max elapsed time))<br>• Visit 2: ENROLMENT (Week 0)<br>• Visit 3: INTERIM Visit (Week 6)<br>• Visit 4: FINAL Visit (Week 12)
**Planned Investigation Period**	Approximately 7 months (inclusive of recruitment and SCREENING period of ~2-3 months)<br><br>Participants involvement will be a maximum of 6 months, inclusive of up to 10 weeks between SCREENING and ENROLMENT.<br><br>Treatment duration per participant is a maximum of 4 months, with a planned treatment duration of 12 weeks and an allowance of +/-3 weeks for each treatment stage post enrolment to allow for scheduling difficulties. It's intended that participants will complete a minimum of 10 weeks treatment.<br><br>Participants receive sound-only stimulation (PS6 – No electrical tongue stimulation (ETS)) during stage 1 (ENROLMENT to INTERIM) and bimodal stimulation (PS6) during stage 2 (INTERIM to FINAL).
**Primary Objective**	The primary objective is to determine whether the addition of tongue stimulation to sound-only stimulation provides additional clinically significant improvement in tinnitus symptoms beyond that of the sound-only stimulation component as measured by the Tinnitus Handicap Inventory (THI).
**Secondary Objective**	To determine the effect of treatment on the symptoms of tinnitus after the addition of tongue stimulation to sound-only stimulation as measured by the Tinnitus Functional Index (TFI).
**Additional Objectives**	To determine the effect of treatment on the quality of life of tinnitus sufferers as measured by the Heath Utilities Index Mark

	III (HUI3) questionnaire and to assess treatment satisfaction based on two satisfaction questions.
**Hypotheses**	The hypothesis for the primary endpoint is that the responder rate during Stage 2 (the second 6-week period of treatment comprising combined sound and tongue stimulation), is greater than the point-estimate of the responder rate observed during Stage 1 (the first 6-week period of treatment comprising sound-only stimulation), where a responder is defined as a participant with an improvement in THI score of at least 7 points.<br>No formal hypothesis testing will be performed for the secondary or additional endpoints of this clinical investigation.
**Primary Endpoint**	The primary endpoint is the responder rate in Stage 2 (the second 6-week period of treatment from INTERIM visit to FINAL visit comprising combined sound and tongue stimulation) compared to the point-estimate of the responder rate observed during Stage 1 (the first 6-week period of treatment from ENROLMENT visit to INTERIM visit comprising sound-only stimulation), where a responder is defined as a participant with an improvement in THI score of at least 7 points.
**Secondary Endpoint**	Changes in symptoms of tinnitus as measured by TFI from the INTERIM visit to the FINAL visit will be reported.
**Additional Endpoints**	Quality of Life/Satisfaction<br>(i) Changes in quality of life as measured by the HUI3 instrument from the SCREENING visit to the INTERIM visit and from the SCREENING visit to the FINAL visit will be reported.<br>(ii) Participant satisfaction rates with treatment as measured by the satisfaction questions at the FINAL visit will be reported.
**Device Name(s)**	*Lenire*® Tinnitus Treatment Device (CE 615889)
**Device Manufacturer**	Neuromod Devices Limited
**Intended Use**	The *Lenire* device is intended to be used by tinnitus sufferers of at least 18 years of age to alleviate the symptoms of chronic (> 3 months), subjective tinnitus.
**Contraindications**	The *Lenire* system is contraindicated for persons:<br>• Who have a pacemaker, defibrillator or any other active implantable device<br>• Who are pregnant, unless directed by a doctor<br>• Who suffer from epilepsy or from any condition that causes loss of consciousness<br>• Who suffer from a condition that causes impaired sensitivity of the tongue<br>• Who have lesions, sores or inflammation of the oral cavity<br>• Who are suffering from any intermittent or chronic neuralgia in the head and neck area.<br><br>*Lenire* has hardware limitations that restrict use in participants with specific pure-tone audiometry hearing characteristics. Specifically, participants with a hearing loss in either ear of 1) greater than 80 dB HL at {2k,3k,4k,6k,8k} Hz, or 2) greater than 40 dB HL at {250,500,1k} Hz should not be fitted with the device.
**Eligibility Criteria**	<u>Inclusion Criteria</u>

	• 18 years and over at time of consent<br>• Ability to read and understand Dutch/Flemish/English or German (depending on the clinical site)<br>• Willing and able to provide and understand informed consent<br>• Willing to commit to the full duration of the investigation<br>• Subjective tinnitus<br>• Tinnitus duration for greater than or equal to 3 months and less than or equal to 10 years at time of consent<br>• Baseline THI greater than or equal to 38<br><br><u>Exclusion Criteria</u><br>• Subjective tinnitus where pulsatility is the dominant feature (participant reported)<br>• Objective tinnitus, where the tinnitus is also observed by the examiner<br>• Commenced usage of hearing aid within the last 90 days<br>• Meniere's disease<br>• Hospitalisation, or visit to a physician, for a head or neck injury, including whiplash, in the previous 12 months<br>• Temporomandibular Joint (TMJ) Disorder<br>• Pregnancy<br>• Oral piercings that cannot or will not be removed for the second stage of the investigation<br>• Neurological condition that may lead to seizures or loss of consciousness (e.g. epilepsy)<br>• Severe cognitive impairment based on Mini-Mental State Examination (MMSE, less than 20)<br>• Participant with a pacemaker or other electro-active implanted device<br>• Abnormal findings following otoscopy/tympanometry that may be contributing to or causing the tinnitus as assessed by an Audiologist/ENT<br>• Initiated new prescription medications or medical treatments in the previous 3 months that may impact the outcomes of the investigation, by discretion of the investigator<br>• Ceased prescription medications or medical treatments in the previous 3 months that may impact the outcomes of the investigation, by discretion of the investigator<br>• State-Trait Anxiety Inventory (STAI) score of >120<br>• Current or previous involvement in medico-legal cases (self-reported).<br>• Participant previously diagnosed with psychosis or schizophrenia<br>• Participants diagnosed with Burning Mouth Syndrome (BMS)<br>• Previous use of *Lenire*<br>• Previous involvement in a clinical investigation for tinnitus or had an experimental/surgical treatment for tinnitus

CONFIDENTIAL: May not be reproduced, distributed or disclosed without prior consent from Neuromod Device Limited

	• Hearing loss of greater than 80 dB HL in any test frequency in the set {2k,3k,4k,6k,8k} Hz or greater than 40 dB HL in the set {250,500,1k} Hz either unilaterally or bilaterally<br>• The site Principal Investigator (PI) does not deem the candidate to be suitable for the investigation for other reasons not listed above. Rationale must be provided.
**Assessments**	Assessments include:<br>• Tinnitus Handicap Inventory (All Visits)<br>• Tinnitus Functional Index (All Visits)<br>• HUI Mark III Assessment (SCREENING, INTERIM and FINAL)<br>• STAI (Screening Tool)<br>• MMSE (Screening Tool)<br>• Audiological Assessment<br>o PTA (SCREENING and FINAL)<br>o Tympanometry (SCREENING, as required by Audiologist at other visits)<br>o Otoscopy (SCREENING, as required by Audiologist at other visits)<br>• Oral Assessment (ENROLMENT and FINAL)<br>• Satisfaction Questions (FINAL)
**Statistical Methods**	**Analysis Populations**<br>The Intent-To-Treat (ITT) population will consist of all participants who meet the eligibility criteria, are enrolled in the investigation and are fitted with the investigational device. ‘Fitted’ is defined as participants receiving a device configured for them, having completed a supervision session and are comfortable taking the device home with them.<br><br>This population will be utilised for the primary analysis of the primary endpoint and the analysis of the secondary and additional endpoints. Missing data will be handled as outlined in the Statistical Analysis Plan.<br><br>The Per-Protocol (PP) population will be defined in the Statistical Analysis Plan. This analysis population will be utilised for the alternative analysis of the primary, secondary and additional endpoints.<br><br>**Statistical Analysis**<br>Continuous variables will be summarised by the number of observations as well as mean, median, standard deviation, minimum, and maximum values. Categorical variables will be summarised using frequencies and percentages. Summaries will be reported for each phase, as appropriate.<br><br>The primary endpoint is p_2, the responder rate during Stage 2 from the INTERIM visit to the FINAL visit (for combined sound and tongue stimulation), compared to p_1, the point-estimate of the responder rate during Stage 1 from the ENROLMENT visit to the INTERIM visit (for sound-only stimulation).

	The null and alternative statistical hypotheses for this endpoint are as follows:<br><br>H_0: $p_2 \leq p_1$<br>H_A: $p_2 > p_1$<br><br>Where a responder is defined as a participant with a clinically meaningful reduction in THI score (at least 7 points) within a treatment stage.<br><br>Hypothesis testing will be performed using a single sample, one-sided normal approximation test (Z-test) for a binomial proportion with a significance level (alpha) of 0.025.<br><br>**Determination of Sample Size**<br>The investigation sample size calculation is based on the primary endpoint hypothesis, i.e. that the responder rate in Stage 2 (the second 6-week period of treatment comprising combined sound and tongue stimulation) is greater than the point-estimate of the responder rate observed during Stage 1 (the first 6-week period of treatment comprising sound-only stimulation), where a responder is defined as a participant with an improvement in THI score of at least 7 points.<br>The sample size calculation was performed with the following assumptions and specifications:<br>• Hypothesis testing will be performed using a single sample, one-sided normal approximation test (Z-test) for a binomial proportion<br>• An estimated responder rate of 45% for p_1 based on relevant data from the previous TENT-A2 study and also accounts for a reasonable upper bound for the placebo effect as observed in the literature.<br>• An estimated responder rate of 60% for p_2 based on relevant data from the previous TENT-A2 study, where using modified Wald binomial probabilities with 90% confidence an estimated responder rate of at least 61% is required and is rounded to 60% to account for a worst case scenario responder rate.<br>• Power $(1-\beta) = 0.8$<br>• Type I error rate $(\alpha) = 0.025$<br><br>These specifications, yielded a sample size estimate of 89 participants to complete all assessments in the clinical investigation.<br><br>The sample size was increased by 20% to allow for a reasonable rate of dropouts or attrition during the study, including due to the current COVID-19 pandemic, and yielded a sample size of up to 112 enrolled participants.

	**Safety Evaluation**<br>All adverse events (AEs), and serious adverse events (SAEs), will be reported through treatment duration of 12 weeks. AEs and SAEs will be further categorised by the relationship to the investigational medical device (Adverse Device Effect, ADE) or an inadequacy of the investigational medical device (device deficiency). AEs will also be reported in relation to the severity of the AEs (‘Mild’, ‘Moderate’ or ‘Severe’ as defined in Section 16.3), as well as the specific onset and offset dates of the AEs. The onset/offset dates will be documented to characterize the transient or permanent nature of the AE’s. The number and percentage of participants with at least one AE will be reported. The THI at final for those participants with an ‘increased tinnitus’ AE still open will also be reported. A full participant listing of all AEs will be provided in the final Clinical Investigation Report. No formal hypothesis testing is planned for the assessment of safety.

CONFIDENTIAL: May not be reproduced, distributed or disclosed without prior consent from Neuromod Device Limited

2.0 Table of Contents

INVESTIGATION REFERENCE NUMBERS 2
[redacted]
1.0 Summary 4
2.0 Table of Contents 10
3.0 Abbreviations 12
4.0 Investigation Plan 15
5.0 Background 15
6.0 Scope 15
7.0 Overview of Clinical Condition 15
7.1 Treatment Options for Tinnitus 16
7.2 Bimodal Neuromodulation for Tinnitus 17
8.0 Overview of Investigational Device 18
8.1 Device Details 18
8.2 Regulatory Status 20
8.3 Intended Use 20
8.4 Contraindications 20
8.5 Medication 21
8.6 Storage Conditions 21
8.7 Device Manufacturer 21
9.0 Investigation Design 21
9.1 Investigation Duration 23
9.2 Investigation Rationale 23
9.3 Intended Purpose of the Investigational Device in the Clinical Investigation 24
[redacted]
10.0 Objectives 27
10.1 Primary Endpoint 27
10.2 Secondary Endpoint 27
10.3 Additional Endpoints 27
11.0 Selection of Sites and Investigators 28
12.0 Population 28
12.1 Number of Participants 28
12.2 Inclusion Criteria 28
12.3 Exclusion Criteria 29
13.0 Visits and Procedures 29
13.1 Schedule of Events 29
13.2 Recruitment 29
13.3 SCREENING Visit 30
13.4 ENROLMENT Visit 30
13.5 Compliance call #1 31
13.6 INTERIM Visit 31
13.7 Compliance call #2 32
13.8 FINAL Visit 32
13.9 Participant Withdrawal 32
13.10 Participant lost-to-follow-up (L2FU) 33

CONFIDENTIAL: May not be reproduced, distributed or disclosed without prior consent from Neuromod Device Limited

13.11 Remote Visits 33
14.0 Data Collection and Analysis 33
14.1 Data Collection 33
14.2 Data Capture and Archival 38
14.3 Database Security 39
14.4 Data Analysis and Statistical Methods 40
14.5 Data Reporting 40
15.0 Statistical Considerations 40
15.1 Sample Size Determination 40
15.2 Populations for Analyses 41
15.3 Statistical Analyses 41
15.4 Primary Endpoint 41
15.5 Secondary Endpoint 42
15.6 Additional Endpoints 42
15.7 Safety Evaluation 43
15.8 Missing Data 43
16.0 Safety Reporting 43
16.1 Definitions 43
16.2 Causality 45
16.3 Severity 47
16.4 Reporting & Recording 47
17.0 Investigation Management 49
17.1 Inspection of Records 49
17.2 Assessment and Management of Risk 50
18.0 Good Clinical Practice and Ethical Considerations 50
18.1 Good Clinical Practice and Regulatory Requirements 50
18.2 Ethics Committee 51
18.3 Informed Consent Procedure 51
18.4 Data Protection and Participant Confidentiality 52
18.5 Investigation Discontinuation and Closure 53
18.6 Other Ethical Considerations 53
18.7 Quality Assurance and Quality Control 53
[redacted]
21.0 References 57
22.0 Appendices 62
Appendix 1 Schedule of Assessments 62
[redacted]

3.0 Abbreviations

AAO-HNSF	American Academy of Otolaryngology—Head and Neck Surgery Foundation
ADE	Adverse Device Effect
AE	Adverse Event
ASADE	Anticipated Serious Adverse Device Effect
BMS	Burning Mouth Syndrome
BRAI3N	Brain Research centre for Advanced, International, Innovative and Interdisciplinary Neuromodulation
BSA	British Society of Audiology
BSI	British Standards Institution
CA	Competent Authority
CBT	Cognitive Behavioural Therapy
CE	Conformité Européenne
CIP	Clinical Investigation Plan
CIR	Clinical Investigation Report
CRA	Clinical Research Associate
CRF	Case Report Form
CRO	Clinical Research Organisation (Avania BV)
dBHL	Decibel Hearing Loss
dBSL	Decibel Sensation Level
DFdiscover	EDC Provider
EC	Ethics Committee
eCRF	electronic Case Report Form
EDC	Electronic Data Capture
ENT	Ear, Nose, and Throat
ETS	Electrical Tongue Stimulation
FAS	Full Analysis Set
FDA	Food and Drug Administration
GDPR	General Data Protection Regulations

GCP	Good Clinical Practice
HUI	Health Utilities Index
IB	Investigator’s Brochure
ICF	Informed Consent Form
ICH	International Council for Harmonisation of Technical Requirements for Pharmaceuticals for Human Use
IFU	Instructions For Use
IOD	Intra-Oral Device
ISO	The International Organization for Standardization
ITT	Intention-To-Treat
kHz	KiloHertz
LDL	Loudness Discomfort Level
LOCF	Last Observation Carried Forward
L2FU	Lost to Follow Up
MCID	Minimal Clinically Important Difference
MDD	Medical Devices Directive (EU) 93/42/EEC
MDR	Medical Devices Regulation (EU) 2017/745
MMSE	Mini Mental State Examination
MS	Multiple Sclerosis
NICE	National Institute for Health and Care Excellence
NIHL	Noise Induced Hearing Loss
PI	Principal Investigator
PIL	Participant Information Leaflet
PMCF	Post-Market Clinical Follow-Up
PMS	Post-Market Surveillance
PP	Per Protocol
PS	Parameter Set
PT	Preferred Term
PTA	Pure-Tone Audiometry
PTM	Progressive Tinnitus Management

CONFIDENTIAL: May not be reproduced, distributed or disclosed without prior consent from Neuromod Device Limited

QoL	Quality of Life
QMS	Quality Management System
RMF	Risk Management File
SADE	Serious Adverse Device Effect
SAE	Serious Adverse Event
SAP	Statistical Analysis Plan
SAR	Statistical Analysis Report
SNHL	Sensorineural Hearing Loss
SOC	System Organ Category
SOP	Standard Operating Procedure
Sponsor	Neuromod Devices Limited
STAI	State Trait Anxiety Inventory
STM	Science Translational Medicine
TENT	Treatment Evaluation of Neuromodulation for Tinnitus
TFI	Tinnitus Functional Index
THI	Tinnitus Handicap Inventory
TMF	Trial Master File
TMJ	Temporomandibular Joint
TRT	Tinnitus Retraining Therapy
TST	Technical Support Team
UIC	Unique Identifier Code
USADE	Unanticipated Serious Adverse Device Effect
21 CFR	Code of Federal Regulations Title 21 [US FDA regulations]

CONFIDENTIAL: May not be reproduced, distributed or disclosed without prior consent from Neuromod Device Limited

4.0 Investigation Plan

The Treatment Evaluation of Neuromodulation for Tinnitus Stage-A3 (TENT-A3) investigation is part of a series of bimodal neuromodulation investigations for the CE marked *Lenire* device. TENT-A3 is a single arm repeated measures prospective investigation.

Participants presenting to one of the study sites with a diagnosis of chronic subjective tinnitus who meet the inclusion/exclusion criteria outlined in Section 12.2 and 12.3 will be enrolled in the investigation while the study site is active.

5.0 Background

The objective of this clinical investigation is to determine whether the addition of tongue stimulation to sound-only stimulation provides additional clinically significant improvements in tinnitus symptoms beyond that of the sound-only stimulation component of the bimodal treatment.

6.0 Scope

The scope of this investigation is to evaluate the contribution of different components of the *Lenire* device on improving tinnitus symptom severity. Neuromod Devices Limited is the legal manufacturer of the *Lenire* device. This product has three components (see Table 1 below), which are collectively referred to as the *Lenire* device throughout the investigation documentation.

7.0 Overview of Clinical Condition

Tinnitus is the perception of sound in the absence of a corresponding external acoustic stimulus. The condition is most commonly referred to as 'ringing in the ears' but symptoms can manifest as buzzing, hissing or clicking. The condition affects approximately 10-15% of the global population [1] [2] [3]. Many tinnitus sufferers report feeling distressed by their symptoms and report a resulting diminishment in their quality of life and that of their families [4]. Those living with tinnitus are further frustrated by their perceived lack of effective treatment options [5]. Tinnitus is heterogeneous with a diverse range of aetiologies, but the most common risk factor is sensorineural hearing loss [6] [7] [8]. One hypothesis is that decreased peripheral auditory activity due to hearing loss causes compensatory changes in firing activity in multiple regions along the ascending auditory and non-auditory pathways that can lead to the tinnitus percept [2] [6] [9] [10] .

In normal hearing individuals, sound travels as vibrations through the outer and middle ears into the cochlea, and cells within the cochlea convert the vibrations into neural signals that transmit along the auditory nerve to the brain [11] [12]. The neural signals travel up through the brainstem, midbrain, and thalamus to the auditory cortex where sound perception occurs. The ascending auditory pathway has a well-organised spatial map of frequencies (i.e., neurons located in a certain region respond best to a specific sound frequency and this spatial ordering of frequencies is known as tonotopy or a tonotopic map). In addition to the ascending pathway, there are dense descending connections from higher auditory and cognitive centres down to earlier stages of auditory neurons, which facilitate sound perception to be modified or fine-tuned by attention, emotion and learning [13-18]. Furthermore, there are widespread

projections from limbic and non-auditory pathways, such as somatosensory pathways, to the auditory network [17] [19-28].

In tinnitus, maladaptive changes in neural firing of one or several regions of the auditory brain may occur [2] [6] [9]. It is hypothesised that the central auditory system overcompensates for the loss of peripheral input and increases the central gain in different networks of neurons along the ascending auditory pathway and in connection with multiple non-auditory brain regions; this overcompensation not only attempts to better sense the incoming sound that leads to excessive cortical activity reaching awareness but also integrates the emotional/distress and cognitive/memory attributes with the phantom percept [29] [30].

7.1 Treatment Options for Tinnitus

Over the past decade the clinical community has sought to coordinate and improve the medical and audiological care provided to tinnitus patients by publishing systematic reviews of available evidence with evidence-based clinical guidelines. In 2014, the American Academy of Otolaryngology—Head and Neck Surgery Foundation (AAO-HNSF) published a Clinical Practice Guideline: Tinnitus [31]. In 2019, A multidisciplinary European guideline for tinnitus: diagnostics, assessment, and treatment was published [32]. In 2020, the National Institute for Health and Care Excellence in the UK published the guideline NG155 Tinnitus: Assessment and Management [33].

The published guidelines describe and consider the evidence for common approaches for managing tinnitus that include:

1. Education and non-specialised counselling
2. Amplification with hearing aids
3. Sound generators to mask or counteract tinnitus perception with or without specialised sound patterns
4. Specialised psychological approaches including cognitive behavioural therapy (“CBT”)
5. Pharmacological approaches
6. Nutritional supplements
7. Combination approaches, e.g., combining sound generators, amplification and/or counselling in a structured manner such as Tinnitus Retraining Therapy (“TRT”) or Progressive Tinnitus Management (“PTM”)
8. Alternative medicine approaches such as acupuncture
9. Transcranial stimulation approaches, e.g., transcranial direct current stimulation and transcranial magnetic stimulation

Only one of these treatment approaches has been reviewed and approved by the US FDA for the intended use of tinnitus treatment: Tinnitus maskers, 510K Classification code KLW.

The published guidelines consistently recommend against pharmacological approaches (e.g., betahistine), nutritional supplements (e.g., ginkgo biloba) and transcranial stimulation because there is either insufficient evidence to recommend, or because there is evidence to

recommend against. There is no recommendation for alternative medicine approaches such as acupuncture.

The guidelines recommend hearing evaluation, with or without cognitive behavioural therapy. Evidence of sufficient quality to support a treatment recommendation was only available to support cognitive behavioural therapy at the time of publication. In recognition of its widespread use by qualified and experienced practitioners, sound therapy is described by AAO-HNSF as an option. NICE concluded that there was insufficient evidence to recommend for or against sound therapy, neuromodulation, and combination approaches and suggested that additional research should be conducted to inform clinical practice decisions.

Despite these consensus guidelines, the diagnosis, treatment and management of tinnitus continue to be suboptimal. Many tinnitus patients continue to suffer because of minimal or inadequate support from their healthcare providers. Two years after the publication of the AAO-HNSF guidelines, a review of common practice concluded that "a vast majority of patients may not be offered management recommendations consistent with the suggested protocol" [34].

7.2 Bimodal Neuromodulation for Tinnitus

To address the unmet clinical need for a safe, effective, and scalable tinnitus treatment, Neuromod developed a non-invasive bimodal (sound and tongue) stimulation device to alleviate the symptoms of chronic, subjective tinnitus. This CE marked device will be used in this investigation and a detailed description of the device is provided in Section 8.

Based on extensive research in animals and several human studies, bimodal neuromodulation using auditory stimulation combined with electrical stimulation of other non-auditory nerves such as the vagus nerve, trigeminal nerve, and somatosensory nerves (e.g., nerves innervating different body regions) has emerged as a promising approach [35-43]. Somatosensory and/or trigeminal inputs can access or modulate neurons throughout the auditory pathway [19] [20] [25] [41] [44-50]. Electrical stimulation of the trigeminal or somatosensory nerves can also activate the ascending reticular activating system of the brain, which consists of neurons involved with emotional, attentional, and cognitive functions [51]. Bimodal stimulation combining electrical stimulation of the trigeminal or somatosensory nerves with sound stimulation can modulate tinnitus-related neuronal activity and potentially drive long-lasting plasticity changes in the brain [52], due to the convergence of inputs into these different auditory and non-auditory regions that can contribute to neural plasticity relevant for tinnitus treatment. Furthermore, such bimodal stimulation approaches can be delivered via portable medical devices suitable for safe and convenient use by patients in the comfort of their home.

CONFIDENTIAL: May not be reproduced, distributed or disclosed without prior consent from Neuromod Device Limited

8.0 Overview of Investigational Device

8.1 Device Details

The *Lenire* device, also referred to as the investigational device throughout this document, is a CE marked medical device intended to reduce the symptoms of tinnitus. It comprises a handheld controller and an intra-oral device called a Tonguetip® Intra-Oral Device (IOD), which delivers gentle electrical stimulation to the tongue, and also comprises of a set of wireless headphones that deliver audio stimulation (See Figure 1). A charger is included that can be used to charge both the controller and the headphones. The sound and tongue stimulation are configured and calibrated to individual participant hearing and sensation characteristics during the initial fitting procedure completed by a trained clinician [see Clinician's Manual (IFU-0026) for further details].

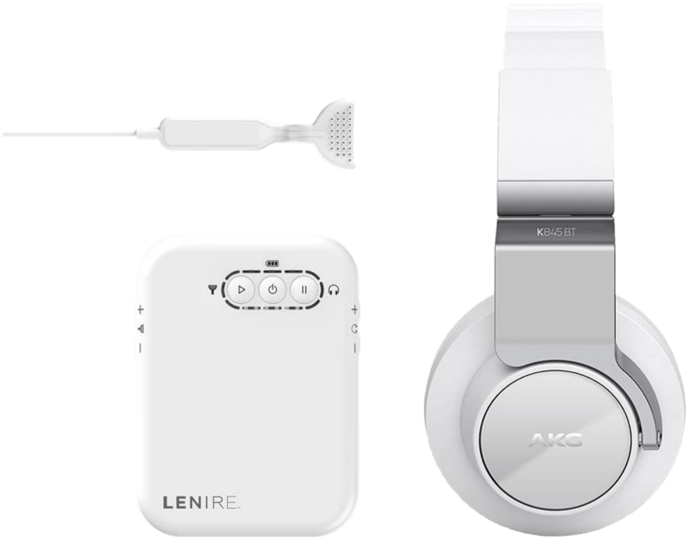

Figure 1: *Lenire* device

[redacted]

[redacted]

[redacted]

8.2 Regulatory Status

The investigational device is CE marked in Europe as a Class IIa medical device. It meets all of the essential requirements of the Medical Device Directive (MDD 93/42/EEC). Approval and certification to Annex II of the MDD (certificate number CE 615889) has been achieved via Neuromod's Notified Body, British Standards Institution (BSI-NL).

Neuromod also holds certification (certificate number MB 615890) for a medical device Quality Management System to ISO 13485:2016, with the scope of "The design, development and manufacture of non-implantable, non-sterile medical devices for the treatment of neurological disorders," also awarded by BSI-NL.

8.3 Intended Use

The *Lenire* device is intended to be used by tinnitus sufferers of at least 18 years of age to alleviate the symptoms of chronic, subjective tinnitus. It should be used for 30 to 60 minutes per day, every day, for at least 10 weeks, after which the participant may experience sustained alleviation of their tinnitus symptoms.The Lenire device can be used in the home environment after consultation and fitting by a suitably qualified clinician. Please refer to the Clinician's Manual [IFU-0027] for further details on device configuration.

Lenire is intended for prescription use only. Use by a person for whom the device has not been configured may cause discomfort, or temporary exacerbation of tinnitus loudness.

8.4 Contraindications

In accordance with the User Manual [IFU-0026]; the *Lenire* system is contraindicated for persons:

- Who have a pacemaker, defibrillator or any other active implantable device.
- Who are pregnant, unless directed by a doctor.
- Who suffer from epilepsy or from any condition that causes loss of consciousness.
- Who suffer from a condition that causes impaired sensitivity of the tongue.
- Who have lesions, sores or inflammation of the oral cavity.
- Who suffer from any intermittent or chronic neuralgia in the head and neck area.

It is essential that these contraindications are communicated to the participant before fitting the device.

Lenire has hardware limitations that restrict use in participants with specific pure-tone audiometry hearing characteristics. Specifically, participants with a hearing loss in either ear of 1) greater than 80 dB HL at {2k,3k,4k,6k,8k} Hz, or 2) greater than 40 dB HL at {250,500,1k} Hz should not be fitted with the device.

CONFIDENTIAL: May not be reproduced, distributed or disclosed without prior consent from Neuromod Device Limited

8.5 Medication

There are no limitations on the use of medications with this device. As is standard clinical practice, the treating healthcare professional will advise participants on any contraindications. See section 12.3 for any medications excluded as part of this investigation.

8.6 Storage Conditions

In accordance with the IFU:

- Storage and transport temperature limits: The lower limit is -20°C and the upper limit is 45°C.
- Storage and transport humidity limits: The lower limit is 5% and the upper limit is 85%.
- Keep dry.
- Fragile, handle with care.

8.7 Device Manufacturer

Neuromod Devices Limited

[redacted]

9.0 Investigation Design

This is a prospective, single arm, repeated measures, multi-site, post-approval investigation of the *Lenire* CE marked medical device to continue to assess the safety and performance of two sequential 6-week stages of different stimulation settings of the investigational device in participants, aged over 18 years, with chronic subjective tinnitus. No masking is applicable given the single arm repeated measures investigation design. The *Lenire* device is expected to be used in accordance with the Instructions For Use (IFU-0026), Clinician's Manual (IFU-0027), CN0072 TENT-A3 Clinical Investigation Plan, Country Specific Participant Information Leaflet and Informed Consent Form, CN0073 Investigator's Brochure (IB) and relevant Standard Operating Procedures (SOPs). The investigation plan has been designed with input from a multi-disciplinary team of clinicians, statisticians, clinical investigation experts and research experts, as is evidenced on the signature page (page 2).

Participants will be recruited through various channels as is standard practice at the investigation site and detailed in the Recruitment Plan. As part of the pre-screening process participants will be asked questions related to the eligibility criteria and may be asked to complete the short-form version of the Tinnitus Handicap Inventory (THI), which

is intended to reduce the burden on the clinic and on participants to avoid a high rate of screen failures. Consent for the collection of pre-screening data and processing to determine potential eligibility will be completed prior to any questionnaires being filled in. The Recruitment Plan provides further detail on this process and the information collected at the pre-screening stage. Participants may also be referred to a SCREENING visit from the standard treatment pathway in the clinic. If participants are deemed to be potentially eligible, they will be invited to the clinic for a SCREENING visit. Before participants undergo any screening assessments for the investigation in the clinic they will go through the informed consent process detailed in Section 18.3. All eligibility questions will be reassessed after the informed consent process is completed. If participants have provided written consent and are deemed eligible for enrolment, they are invited to an ENROLMENT visit where they are trained and fitted with the device. ENROLMENT will take place no more than 10 weeks after the initial SCREENING visit. The treatment consists of one device per participant and is typically delivered at home by the participant. Participants will be asked that treatment is completed for one hour per day, in two 30 minute sessions every day, for the 12 week period.

The recommended schedule is to have an INTERIM visit (Visit 3) approximately 6 weeks after ENROLMENT (Visit 2). The parameter set is updated from PS6-No ETS to PS6 at this visit and compliance data checked on the device. The FINAL visit (Visit 4) should be scheduled approximately 12 weeks after ENROLMENT. Unscheduled visits for withdrawal from the investigation or if required for adverse events can be scheduled at any time from ENROLMENT to the FINAL visit. The main assessment, Tinnitus Handicap Inventory (THI), is captured at every timepoint (including at withdrawal visits if possible). Participants will receive two calls during the investigation to encourage treatment compliance, where the first call will occur between the ENROLMENT and INTERIM visits and the second call will occur between the INTERIM and FINAL visits (Please refer to Section 13 for more detailed information on the visits).

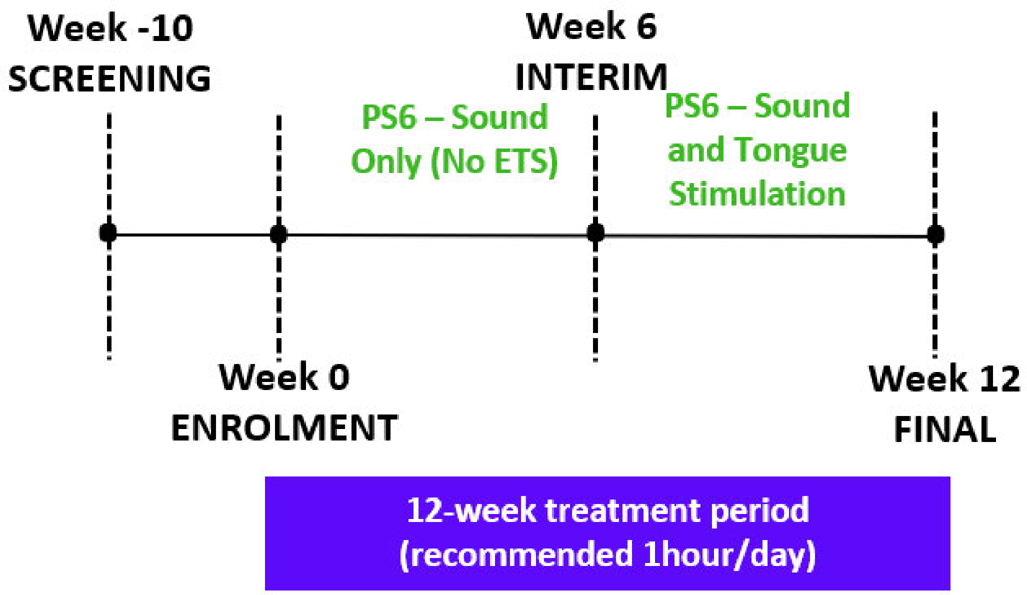

Figure 3: Investigation Flowchart.

9.1 Investigation Duration

The investigation duration is expected to be approximately 7 months to allow for recruitment, screening and the 3-month treatment period. All enrolled participants are expected to complete 3 months of treatment with the *Lenire* device. The investigation will be completed when all enrolled participants have completed their treatment or have been identified as 'lost to follow-up'.

9.2 Investigation Rationale

This is a single-arm repeated measures investigation that aims to build on earlier evidence (outlined below) to further evaluate the additional clinical effect of adding tongue stimulation to sound-only stimulation and to continue to confirm the safety and performance of the *Lenire*® device.

In 2020, results from the largest randomized and blinded clinical investigation for bimodal neuromodulation in tinnitus (TENT-A1 investigation; clinicaltrials.gov: NCT02669069) with 326 enrolled participants were published in Science Translational Medicine [52]. These results supported the safety and efficacy of combined sound and tongue stimulation therapy with the *Lenire* device across three different stimulation parameter settings (PS1, PS2, PS3) [52]. The TENT-A1 investigation reported that bimodal neuromodulation provided clinical benefit for about two-thirds of participants during the 12-week treatment period and achieved significant reductions in tinnitus symptoms that could last for 12 months after the treatment ended. There were no treatment related serious adverse events throughout the investigation and there were high treatment compliance rates (84%) and satisfaction rates (67%) across investigation participants.

Based on the findings from the TENT-A1 investigation, a subsequent investigation, referred to as TENT-A2 (Treatment Evaluation of Neuromodulation for Tinnitus – Stage 2; clinicaltrials.gov: NCT03530306), was carried out to investigate and answer several questions that arose from the TENT-A1 investigation including the effects of different sound and tongue stimulation components on therapeutic outcomes and the effects of adjusting different stimulation parameters or components to further improve tinnitus symptoms over time. The study consisted of four treatment arms with different parameter settings or components evaluated over a 12-week treatment period with a post-treatment follow-up period of 12 months.

TENT-A2 showed that changing the stimulation settings over time provided enhanced clinical benefits to participants. The majority of participants exhibited a clinically meaningful reduction in symptoms during the first 6-weeks, which was further reduced during the second 6-weeks of treatment by changing the stimulation settings. There were no significant differences between arms, suggesting that the exact parameters of the acoustic stimuli did not appear to be responsible for driving the therapeutic effect in treating tinnitus. The investigation also found that adding electrical tongue stimulation to sound-only stimulation during a second 6-week period after an initial 6-week period of sound-only stimulation resulted in additional therapeutic benefit. As in the TENT-A1 investigation, there were no treatment related serious adverse events in the TENT-A2 investigation and

there were high treatment compliance and satisfaction rates across investigation participants.

The findings from the TENT-A2 investigation support the importance of combining tongue stimulation with sound stimulation for tinnitus treatment with the *Lenire* device; however, a multi-site study in a larger cohort of participants than in the previous TENT-A2 investigation will more fully inform our understanding of the additional benefit conferred by the tongue stimulation component of the *Lenire* device.

1) Neuromod aims to confirm that the addition of tongue stimulation to sound-only stimulation provides additional clinically significant improvements in tinnitus symptoms beyond that of the sound component of the *Lenire* tinnitus treatment by means of prospective data collection using the same bimodal treatment parameter set [known as PS6] and its equivalent sound-only stimulation [PS6 - no ETS] as used in Arm 4 of the previous TENT-A2 investigation. Note that the previous TENT-A2 investigation evaluated different parameter settings for bimodal treatment [including PS6] and for sound-only stimulation [previously known as PS9]. The sound stimulation of PS9 is the exact same as that used in PS6. The safety and performance of the device will continue to be monitored. The investigation design and data compiled from the investigation will be part of Neuromod's Clinical Evaluation Plan.
2) This current investigation also aims to show the generalisability of the previous TENT-A2 investigation outcomes to a larger cohort of participants and across multiple sites. The current investigation is being performed at different clinical sites led by separate investigators in different countries, which further contributes to the generalisability of the results.

Positive findings from the current investigation will not only confirm the findings from the TENT-A2 investigation, but will also support the generalisability of the therapeutic outcomes to a much larger number of participants across different clinical sites.

9.3 Intended Purpose of the Investigational Device in the Clinical Investigation

Lenire is a non-surgical medical device designed to reduce the symptom severity of subjective tinnitus and will be used in accordance with the intended use set out in the CE certification.

[redacted]

[redacted]

CONFIDENTIAL: May not be reproduced, distributed or disclosed without prior consent from Neuromod Device Limited

[redacted]

CONFIDENTIAL: May not be reproduced, distributed or disclosed without prior consent from Neuromod Device Limited

10.0 Objectives

Primary Objective

The primary objective is to determine whether the addition of tongue stimulation to sound-only stimulation provides additional clinicially significant improvement in tinnitus symptoms beyond that of the sound-only stimulation component as measured by the Tinnitus Handicap Inventory (THI).

Secondary Objective

To determine the effect of treatment on the symptoms of tinnitus after the addition of tongue stimulation to sound-only stimulation as measured by the Tinnitus Functional Index (TFI).

Additional Objectives

To determine the effect of treatment on the quality of life of tinnitus sufferers as measured by the Heath Utilities Index Mark III (HUI3) questionnaire and to assess treatment satisfaction based on two satisfaction questions.

10.1 Primary Endpoint

The primary endpoint is the responder rate in Stage 2 (the second 6-week period of treatment from INTERIM visit to FINAL visit comprising combined sound and tongue stimulation) compared to the point-estimate of the responder rate observed during Stage 1 (the first 6-week period of treatment from ENROLMENT visit to INTERIM visit comprising sound-only stimulation), where a responder is defined as a participant with an improvement in THI score of at least 7 points.

10.2 Secondary Endpoint

The secondary endpoint is to assess changes in symptoms of tinnitus as measured by TFI from the INTERIM visit to the FINAL vist**.**

10.3 Additional Endpoints

The following additional endpoints are included to assess the effects of treatment on quality of life participants as well as overall participant satisfaction with treatment:

Quality of Life/Satisfaction

(i) Changes in quality of life as measured by the HUI3 instrument from the SCREENING visit to the INTERIM visit and from the SCREENING visit to the FINAL visit will be reported.
(ii) Participant satisfaction rates with treatment as measured by the satisfaction questions at the FINAL visit will be reported.

11.0 Selection of Sites and Investigators

Site	PI
See Appendix 4 for a list of sites	See Appendix 4 for a list of investigators<br>Roles and Responsibilities to be outlined in Signature and Delegation Log

12.0 Population

Adults (at least 18 years of age) with subjective, chronic tinnitus (≥3 years and ≤10 years) and with a THI score of ≥ 38 points.

The intended population for this device is largely representative of the population to be included in this investigation. To ensure that standard of care for tinnitus sufferers is closely replicated in this current investigation, the exclusion criteria also includes those comorbidities often representative in a tinnitus population that may need to be addressed prior to commencing tinnitus treatment with *Lenire* but are not contraindicated for the device.

12.1 Number of Participants

It has been calculated that 89 participants are expected to complete the investigation to ensure that the investigation is powered appropriately. The sample size was increased by 20% to allow for a reasonable rate of dropouts or attrition during the study, including due to the current Covid-19 pandemic, and yielded a sample size of up to 112 enrolled participants.

Investigation eligibility will be confirmed by the site team from medical information provided by the participant. Prior to treatment with *Lenire*, the PI (or appropriately designated personnel) will ensure that treatment with *Lenire* is not contraindicated in accordance with the IFU and the eligibility criteria outlined below.

Ineligible participants will be offered clinical care as per standard clinical practice at the site.

12.2 Inclusion Criteria

- 18 years and over at time of consent
- Ability to read and understand Dutch/Flemish/English or German (depending on the site)
- Willing and able to provide and understand informed consent
- Willing to commit to the full duration of the investigation
- Subjective tinnitus
- Tinnitus duration for greater than or equal to 3 months and less than or equal to 10 years at time of consent
- Baseline THI greater than or equal to 38

12.3 Exclusion Criteria

- Subjective tinnitus, where pulsatility is the dominant feature (participant reported)
- Objective tinnitus, where the tinnitus is also observed by the examiner
- Commenced usage of hearing aid within the last 90 days
- Meniere's disease
- Hospitalisation, or visit to a physician, for a head or neck injury, including whiplash, in the previous 12 months
- TMJ Disorder
- Pregnancy
- Oral piercings that cannot or will not be removed for the second stage of the investigation
- Neurological condition that may lead to seizures or loss of consciousness (e.g. epilepsy)
- Severe cognitive impairment based on MMSE (score less than 20)
- Participant with a pacemaker or other electro-active implanted device
- Abnormal findings following otoscopy/tympanometry that may be contributing to or causing the tinnitus as assessed by an Audiologist/ENT
- Initiated new prescription medications or medical treatments in the previous 3 months, that may impact the outcomes of the investigation, by discretion of the investigator
- Ceased prescription medications or medical treatments in the previous 3 months that may impact the outcomes of the investigation, by discretion of the investigator
- STAI score of >120
- Current or previous involvement in medico-legal cases (self-reported)
- Participant previously diagnosed with psychosis or schizophrenia
- Participants diagnosed with BMS
- Previous use of *Lenire*
- Previous involvement in a clinical investigation for tinnitus or had an experimental/surgical treatment for tinnitus
- Hearing loss of greater than 80 dB HL in any test frequency in the set {2k,3k,4k,6k,8k} Hz or greater than 40 dB HL in the set {250,500,1k} Hz either unilaterally or bi-laterally
- The site PI does not deem the candidate to be suitable for the investigation for other reasons not listed above. Rationale must be provided

13.0 Visits and Procedures

13.1 Schedule of Events

See Appendix 1 for schedule of events.

13.2 Recruitment

Based on standard clinical practice at the site, participants eligible for treatment with *Lenire* will be potentially eligible for inclusion in the investigation. If the eligibility criteria

CONFIDENTIAL: May not be reproduced, distributed or disclosed without prior consent from Neuromod Device Limited

(Section 12.2 and Section 12.3) that can be assessed as part of pre-screening (as detailed in the Recruitment Plan) appear to be met, the participant will be informed of the investigation by the investigation team, who will provide them with the investigation information as per local procedures, such as in person, via an online portal, email or through the post. If the participant is interested, they will be invited to a SCREENING visit to assess eligibility for the investigation. Participants may express their interest in participation:

- in person at the time of receiving investigation information
- during a follow-up call from delegated site team members
- by telephoning the site team
- by responding to email or online portal message

Participants may also be recruited through various channels as is standard practice at the investigation site such as advertising for the trial on online forums or via online/radio advertisements as per the Recruitment Plan. Particpants may be required to fill out a pre-screening form to determine possible eligibility for the investigation. Recruitment material will be provided to and approved by ethics prior to the commencement of recruitment for the investigation. GDPR consent for the collection and processing of data to determine potentially eligible particpants will be taken prior to any questionnaires being completed. The Recruitment Plan details the eligibility questions to be asked as part of the pre-screening process.

Information regarding the investigation will be provided prior to or on the day of the SCREENING Visit. Informed consent will be completed at the SCREENING visit prior to any assessments being carried out. Participants who are not suitable for treatment with *Lenire* may avail of treatment as per local standard of care at the site.

13.3 SCREENING Visit

Screening assessments are to be completed as per the Schedule of Events in Appendix 1. The SCREENING visit will be used to perform an eligibility check on participants that are interested in taking part in the trial. A hearing assessment will be completed as part of the eligibility assessment and later used to configure the investigational device if a participant is enrolled on the trial.

Consent will be obtained by delegated team members as outlined in Section 18.3 at this visit prior to any investigation assessments being performed.

13.4 ENROLMENT Visit

Prior to being enrolled in the investigation, the Principal Investigator (or appropriately designated personnel) will re-confirm that treatment with *Lenire* is not contraindicated in accordance with the IFU. Participants are deemed to be enrolled in the investigation if they have signed informed consent, have met the eligibility criteria and are fitted with the device at the ENROLMENT visit. 'Fitted' is defined as participants receiving a device configured for them, having completed a supervision session and are comfortable taking the device home with them.

CONFIDENTIAL: May not be reproduced, distributed or disclosed without prior consent from Neuromod Device Limited

Enrolment assessments will be completed as per the Schedule of Events in Appendix 1. Participants will be given comprehensive training on use of the investigational device during the ENROLMENT visit by an appropriately trained member of the site team. Training includes instructions on investigational device usage and review of the User Manual. Each device is supplied to the participant with a User Manual and Quick Start Guide. Participants will be provided with information on how to address common technical issues that could arise and how to contact technical support. Participants will complete a supervised treatment session of approximately 5-15 minutes duration with the device that has been calibrated and configured for their use to ensure they are comfortable using the device.

[redacted]

Data will be collected on an Enrolment case report form (CRF) in the Electronic Data Capture (EDC) system (DFdiscover) designed specifically for this investigation, as outlined in Section 14.0.

13.5 Compliance call #1

To help ensure compliance, participants will receive one scheduled phone call to remind them to use their investigational device and return for site visits. The compliance calls will occur between ENROLMENT and INTERIM visits.

Any adverse events reported should be treated as per local standard of care and recorded on the AE CRF.

13.6 INTERIM Visit

It is required that participants complete the INTERIM visit approximately 6 weeks after their initial appointment. There is an allowance of +/- 3 weeks for the visit to be completed to allow for scheduling conflicts. The assessments as outlined in Appendix 1 will be performed.

[redacted]

Compliance data may be downloaded from the usage log on the participant's investigational device and reviewed by the investigator. Usage will be discussed with the participant and they will be given the opportunity to discuss any technical issues

they may be experiencing with the investigational device. Any technical issues experienced throughout the trial will be logged as a device deficiency and trended appropriately. Technical support will issue replacement parts if required. The investigational device logs the time, date and the duration of each treatment session and stimulus intensities.

Data will be collected on an INTERIM CRF on the EDC system, as outlined in Section 14.0.

Any adverse events reported should be treated as per local standard of care and recorded on the AE CRF.

13.7 Compliance call #2

To help ensure compliance, participants will receive one scheduled phone call to remind them to use their investigational device and return for site visits. Compliance calls will occur between INTERIM and FINAL visits.

Any adverse events reported should be treated as per local standard of care and any AEs recorded on the AE CRF.

13.8 FINAL Visit

It is required that participants complete the FINAL visit approximately 6 weeks after theINTERIM appointment. There is an allowance of +/- 3 weeks for the visit to be completed to allow for scheduling conflicts. The assessments as outlined in Appendix 1 will be performed. Data will be collected on the Final Assessment CRF, as outlined in Section 14.0.

An oral and audiological assessment will be conducted as part of the FINAL visit to document the oral and aural health of all participants, respectively. The participants are expected to bring their investigational device to this visit so that the site can retrieve compliance data.

Adverse events (refer to Appendix 2) should be treated as per local standard of care and any AEs recorded on the CRF.

[redacted]

13.9 Participant Withdrawal

Participants are free to leave the investigation at any time and without giving a reason. Records relating to treatment will be kept, as this is valuable to the investigation. Information on how to withdraw will be provided in the Participant Information Leaflet.

CONFIDENTIAL: May not be reproduced, distributed or disclosed without prior consent from Neuromod Device Limited

Participants may stop using the device at any time or when instructed by the clinical provider to do so, e.g., if an adverse event occurs.

A decision to withdraw at any time, or a decision not to take part, will not affect the their ability to receive standard of care at the local site or alternative clinics.

Investigators can withdraw a participant if they become aware of any new information that contraindicates a participant for use of the device (e.g., becoming pregnant during the investigation).

13.10 Participant lost-to-follow-up (L2FU)

Participants will be sent a reminder correspondence (in accordance with local standard of care) e.g., via email, text or online portal for clinic visits. If participants do not attend their scheduled visit, a further reminder and alternative appointment will be scheduled. If participants do not return for their alternative scheduled visit, any reasons provided for not attending will be recorded and participants will be deemed lost to follow-up. Participants that are lost-to-follow-up or withdraw from the investigation will not be replaced. All attempts to contact the participant will be documented in order to verify contact attempts. After a minimum of 3 documented contact attempts, a participant can be considered lost to follow-up.

13.11 Remote Visits

To facilitate the current COVID-19 pandemic where travel restrictions are in a state of flux, the possibility for remotely collecting the data via a telephone or suitably assessed teleconference system may be required. If remote visits are required, a courier service will be selected to collect devices and deliver them to the clinic to update stimulation settings. Once the necessary updates are made, the device will be couriered back to the participants preferred mailing address as remote updates are currently not available on the device.

Remote visits are not the preferred option to collect the data; however, if remote visits are required, at a minimum the collection of the primary endpoints and adverse event related data will be prioritised.

14.0 Data Collection and Analysis

14.1 Data Collection

The following assessments and methods will be utilised in order to gather data for this investigation. The data points collected provide input for each of the performance and safety endpoints detailed in Section 10. Any equipment required to complete audiological assessments will require evidence of calibration certs.

- **Medical History**
  - Tinnitus history: location or side of perceived tinnitus, tinnitus duration, previous tinnitus treatments, type and sound of tinnitus
  - Other medical conditions

- Concomitant prescription medication
- Demographics (gender, age, race, ethnicity, employment status, current/previous member of military)
- Questions relating to investigation inclusion and exclusion criteria

Updated medical history will be captured at each visit, including changes in tinnitus characteristics or any noticeable differences in hearing. New medical treatments or prescribed medication since the previous visit will also be captured on the updated medical history form.

- **Tinnitus Handicap Inventory (THI)**

  The Tinnitus Handicap Inventory (THI) is a validated psychometric questionnaire used to determine the severity of tinnitus symptoms and is one of the most widely established instruments for assessing tinnitus symptom severity [53]. The reliability of the THI has previously been demonstrated, and it has been validated in many languages [54].

  The THI is comprised of 25 questions, which can be answered as “No,” “Yes,” or “Sometimes,” to which a numerical score of 0, 4, or 2 is assigned, respectively. The total THI score is the sum of all item scores and can range from 0 (“no handicap”) to 100 (“catastrophic handicap”). The THI scores can also be categorised into five severity levels of tinnitus handicap; slight (0-16), mild (18-36), moderate(38-56), severe(58-76) and catastrophic (78-100). The minimal clinically important difference (MCID) reported for THI is 7 points [55] and represents a clinically meaningful change in tinnitus symptoms as described in a review of the relevant literature prepared by Prof. Berthold Langguth for the FDA as part of the discussions related to Q190408/S001 Clinical supplement to Pre-Sub meeting request [Appendix 3].

  A THI questionnaire will be completed by the participant at each visit. The THI result will be inputted into the CRF.

- **Investigational Device**

  Device Fitting: Participant’s pure-tone audiometric thresholds (250 Hz to 8 kHz) will be measured at the SCREENING visit and subsequently used to configure the sound stimuli to just above their hearing threshold at each tone frequency. The participant can adjust the default sound stimulus loudness (amplification) between -12 dB and +12 dB during treatment using volume buttons on the controller. [redacted]

[redacted] The treatment device reverts to the default intensities at the start of each new session and all changes to stimulation settings are recorded on the device log.

Participants are fitted for their device at the ENROLMENT visit. 'Fitted' is defined as participants receiving a device configured for them, having completed an approximately 5-15 minute supervision session and are comfortable taking the device home with them.

- **Compliance:** The *Lenire* device records the times and duration of use to an inbuilt memory chip. When the participant brings their device to the site, this usage data will be extracted and may be used for subsequent per-protocol analysis. This data will provide information regarding device usage and compliance with treatment plan. Participants will be classified as either compliant or non-compliant to treatment plan, where the former is defined as at least 18 hours of device usage between ENROLMENT and INTERIM as well as at least 18 hours of device usage between INTERIM and FINAL visits.

- **Device Traceability:** Each device has a serial number which will be recorded on participant files and CRFs. In the event of an issue with the device, the investigational device should be returned to the manufacturer according to standard site and device manufacturer safety reporting procedures. See Section 16.0 for further details on safety reporting.

- **Stimulation Parameter Sets:** Each participant will receive the sound-only stimulation parameter set [PS6 – no ETS] for the first six weeks of treatment. At the INTERIM visit, the stimulation will be updated using the *Lenire* Fitting Software that investigators have previously received training on. The stimulation is updated to PS6. There is no difference to the sound stimulation component received by participants but they will now use the Tonguetip IOD component of the device.

[redacted]

- **Adverse Events**
  Reported adverse events [defined in Section 16] will be systematically recorded for the 12 week treatment period (from ENROLMENT to FINAL). Participants typically report adverse events during clinical assessments, during telephone calls or by emailing the site team. Adverse events observed by the PI or treating team will be systematically recorded throughout the investigation.

  Unresolved device related adverse events will be followed-up after the end of the investigation. Participants with these unresolved AEs will be asked to consent to allow for the data to be shared with the Sponsor and regulatory authorities, where applicable.

- **Satisfaction Questions**
  Participants will be asked to complete two questions to provide their feedback on their satisfaction of the treatment at the FINAL visit.

- **HUI Mark III**
  The Health Utilities Index Mark III (HUI3) is a quality of life assessment with 17 questions. HUI® provides descriptive evidence on multiple dimensions of health status, a score for each dimension of health, and a health-related quality of life (HRQL) score for overall health. The utility scores have interval-scale properties. Overall HRQL scores are on the conventional 0-1 scale where 1 depicts perfect health and are appropriate for calculating quality-adjusted life years (QALYs) in cost-effectiveness and cost-utility analyses [56]. This assessment will be completed at SCREENING, INTERIM and FINAL.

- **State-Trait Anxiety Inventory**
  The State-Trait Anxiety Inventory (STAI) is a psychological inventory based on a 4-point Likert scale [57]. It consists of 40 self-reported questions and measures two types of anxiety: state anxiety (anxiety about an event) and trait anxiety (anxiety level as a personal characteristic). Higher scores are positively correlated with higher levels of anxiety. The cut-off of 120 is as per the previous TENT studies exclusion criteria. This is a screening tool that is only assessed at the SCREENING visit.

- **Mini Mental State Examination**
  The Mini-Mental State Exam (MMSE) is a widely used instrument for screening for cognitive impairment [58]. The total score is out of 30. A score of 20 or below indicates severe cognitive impairment [59]. This is a screening tool that is only assessed at the SCREENING visit.

- **Oral Health Assessment**
  The Oral Health Assessment is a visual inspection of the oral cavity and general mouth area. This assessment is completed at ENROLMENT to determine whether a participant has lesions, sores or inflammation of the oral cavity as these are contraindicated. If the participant presents with any lesions, sores or inflammation, they will be instructed not to start treatment until the issue has resolved. The participant should contact the site team so that they are aware when treatment has started and the INTERIM visit can be scheduled accordingly. An oral assessment will also be conducted as part of the FINAL visit to document the oral health of all participants.

- **Pure Tone Audiometry**
  Air-conduction Pure Tone Audiometry (PTA) will be conducted binaurally to assess participants' hearing thresholds pre- and post- treatment in accordance with the procedure outlined by the British Society of Audiology [60]. Measurements will be made at the standard audiometric test frequencies {250, 500, 1k, 2k, 3k, 4k, 6k and 8k} Hz using standard audiometry equipment. The PTA will be assessed at the SCREENING and FINAL visits. The Audiologist may carry out the assessment at other visits if required.

CONFIDENTIAL: May not be reproduced, distributed or disclosed without prior consent from Neuromod Device Limited

- **Tympanometry**
  Tympanometry is an objective test that measures the movement and function the middle ear and eardrum (tympanic membrane) [61]. Tympanometry will be conducted at SCREENING. The Audiologist may carry out the assessment at other visits if required.

- **Otoscopy**
  Otoscopy is a clinical procedure used to examine structures of the ear, particularly the external auditory canal, tympanic membrane, and middle ear [62]. Otoscopy will be conducted at SCREENING. The Audiologist may carry out the assessment at other visits if required.

- **Tinnitus Functional Index**
  The Tinnitus Functional Index **(**TFI) is a psychometric questionnaire used to determine the severity of tinnitus symptoms. It employs a 10-point Likert scale for responses [63]. A TFI questionnaire will be completed by the participant at each visit The TFI result will be inputted into the CRF.

14.2 Data Capture and Archival

Data will be captured in electronic format on an electronic data capture [EDC] system specifically adapted for the investigation. The EDC system, DFdiscover, will be managed and maintained by the clinical research organisation contracted to oversee the investigation, Avania BV. DFdiscover is 21 CFR Part 11 compliant and will not contain any participant identifiable information. The database will be designed and maintained in accordance with local data protection laws. An audit trail is available for tracking all information that the EDC user enters, modifies or deletes.

The investigators shall ensure the accuracy, completeness, legibility and timelines of the data reported in eCRF (electronic Case Report Forms) and in all required documentation. Where the eCRFs are individual forms captured using the EDC system. Data reported on the eCRF shall be supported by the source documents, with exceptions outlined below, with any discrepancies being explained. Any corrections made to documents will be done according to ISO 14155 guidelines. If an item is not available or is not applicable, this fact should be indicated; no space is to be left blank. The investigator who has signed the clinical investigation plan signature page or his/her authorized designee is to personally sign the eCRFs to validate that the observations and findings are recorded on the eCRFs correctly and completely. The eCRFs are to be completed in full, in a timely manner, after the participant’s visit. Failure to meet the documentation requirements may lead to the disqualification of an investigator.

Data will be directly entered into the EDC system where practically possible or transferred from the electronic health record or audiometry software where applicable. Questionnaires and eligibility assessment at SCREENING will be entered directly into the EDC system. Oral assessment data will also be entered directly into the EDC system.

CONFIDENTIAL: May not be reproduced, distributed or disclosed without prior consent from Neuromod Device Limited

Case Report Forms (CRFs) will be collected/completed for each participant at each visit. Only electronic CRFs (eCRFs) will be completed for this investigation. Participant medical records and electronic assessments (i.e., eCRFs entered directly into the EDC or collected via other electronic sources) will be considered source data. Where data is directly entered into the EDC system, the EDC will be considered source data.

The database will be stored safely and securely in accordance with local data protection laws for the duration of the investigation and thereafter by the Sponsor/CRO. Access to the site medical records will be provided to the clinical research associate (CRA) at in-person or remote monitoring visits (following the appropriate remote monitoring guidelines). The CRA will have remote access to the EDC for the duration of the investigation and database close out.

The data entered into the EDC will be fully validated, using clinical investigation-specific ranges and consistency checks and database listings. Queries will be issued to the site via the EDC system, and are to be resolved by the investigator or their designee using the EDC system. Data validation will be completed on a regular basis. The entire database will be re-validated to ensure that there are no outstanding data discrepancies prior to database lock. Any changes to the database after that time will require written agreement by the Sponsor.

14.3 Database Security

Data protection will be ensured in accordance with Regulation (EU) 2016/679 (General Data Protection Regulation, GDPR). This includes the following:

Personal data shall be:

- processed lawfully, fairly and in a transparent manner in relation to the data subject (‘lawfulness, fairness and transparency’);
- collected for specified, explicit and legitimate purposes and not further processed in a manner that is incompatible with those purposes; further processing for archiving purposes in the public interest, scientific or historical research purposes or statistical purposes shall, in accordance with Article 89(1), not be considered to be incompatible with the initial purposes (‘purpose limitation’);
- adequate, relevant and limited to what is necessary in relation to the purposes for which they are processed (‘data minimisation’);
- accurate and, where necessary, kept up to date; every reasonable step must be taken to ensure that personal data that are inaccurate, having regard to the purposes for which they are processed, are erased or rectified without delay (‘accuracy’);
- kept in a form which permits identification of data subjects for no longer than is necessary for the purposes for which the personal data are processed; personal data may be stored for longer periods insofar as the personal data will be processed solely for archiving purposes in the public interest, scientific or historical research purposes or statistical purposes in accordance with Article 89(1) subject to implementation of the appropriate technical and organisational measures

required by this Regulation in order to safeguard the rights and freedoms of the data subject (‘storage limitation’);

- processed in a manner that ensures appropriate security of the personal data, including protection against unauthorised or unlawful processing and against accidental loss, destruction or damage, using appropriate technical or organisational measures (‘integrity and confidentiality’).

14.4 Data Analysis and Statistical Methods

See Section 15.0 Statistical Considerations.

14.5 Data Reporting

The data will be analysed in accordance with the Statistical Analysis Plan. A final report will be completed and sent to ethics in accordance with the reporting requirements set out in ISO14155:2020 after Last Participant Last Visit and all the necessary data close out activities have been completed. Findings from this investigation will be taken into account for the post market surveillance and the risk management file (RMF) that form part of the device technical documentation to obtain and maintain a CE-mark. The results will be disseminated through peer-reviewed journals.

15.0 Statistical Considerations

15.1 Sample Size Determination

The investigation sample size calculation is based on the primary endpoint hypothesis that the responder rate in Stage 2 (the second 6-week period of treatment comprising combined sound and tongue stimulation) is greater than the point-estimate of the responder rate observed during Stage 1 (the first 6-week period of treatment comprising sound-only stimulation), where a responder is defined as a participant with an improvement in THI score of at least 7 points.

The sample size calculation was performed with the following assumptions and specifications:

- Hypothesis testing will be performed using a single sample, one-sided normal approximation test (Z-test) for a binomial proportion.
- The null hypothesis is that the percentage of participants achieving at least 7 points reduction in THI score in Stage 2 (p_2) is less than or equal to the point-estimate of the responder rate in Stage 1 (p_1; H_0: $p_2 \leq p_1$).
- The alternative hypothesis is that the percentage of participants achieving at least 7 points reduction in THI score in Stage 2 (p_2) is greater than the point-estimate of the responder rate in Stage 1 (p_1; Ha: $p_2 > p_1$).
- An estimated responder rate of 45% for p_1 is based on relevant data from a previous TENT-A2 study and also accounts for a reasonable upper bound for the placebo effect as observed in the literature.
- An estimated responder rate of 60% for p_2 is based on relevant data from a previous TENT-A2 study, where using modified Wald binomial probabilities with 90% confidence for an estimated responder rate of at least 61% is

required and is rounded to 60% to account for a worst case scenario responder rate.

- Power (1- β) = 0.8.
- Type I error rate (α) = 0.025.

These specifications, yielded a sample size estimate of 89 participants to complete all assessments in the clinical investigation.

The sample size was increased by 20% to allow for a reasonable rate of dropouts or attrition during the study, including due to the current COVID-19 pandemic, and yielded a sample size of up to 112 enrolled participants.

[redacted]

15.2 Populations for Analyses

The Intent-To-Treat (ITT) population will consist of all participants who meet the eligibility criteria, are enrolled in the investigation and are fitted with the investigational device. ‘Fitted’ is defined as participants receiving a device configured for them, having completed a supervision session and are comfortable taking the device home with them.

This population will be utilised for the primary analysis of primary endpoint and the analysis of the secondary and additional endpoints. Missing data will be handled as per the SAP.

The Per-Protocol (PP) population will be defined in the SAP. This analysis population will be utilised for the alternative analysis of the primary, secondary and additional endpoints.

15.3 Statistical Analyses

Continuous variables will be summarised by the number of observations as well as mean, median, standard deviation, minimum, and maximum values. Categorical variables will be summarised using frequencies and percentages. Summaries will be reported for each phase, as appropriate.

Additional details regarding the performance and safety variable definitions, analyses strategy, statistical justification, and techniques for handling missing values will be detailed in the SAP. In the SAP, procedures for reporting any deviation(s) from the SAP will also be described.

15.4 Primary Endpoint

The primary endpoint is the responder rate (percentage of participants achieving at least 7 points reduction in THI score) in Stage 2 of the study (from the INTERIM visit to the FINAL visit), attributed to the addition of tongue stimulation to sound-only

CONFIDENTIAL: May not be reproduced, distributed or disclosed without prior consent from Neuromod Device Limited

stimulation, compared to the point-estimate of the responder rate in Stage 1 (from the ENROLMENT visit to INTERIM visit) for sound-only stimulation. The null and alternative statistical hypotheses for this endpoint are as follows:

$$H_0: p_2 \leq p_1$$

$$H_A: p_2 > p_1$$

Where $\mathbf{p_1}$ is the responder rate observed in Stage 1 (sound-only stimulation; the performance goal), and $\mathbf{p_2}$ is the responder rate in Stage 2 (addition of tongue stimulation to sound-only stimulation), where a responder is defined as a participant with clinically meaningful reduction in THI score (of at least 7 points) within the corresponding treatment stage. The null hypothesis will be tested using a single sample, one-sided normal approximation test (Z-test) for a binomial proportion with a significance level of 0.025.

[redacted]

15.5 Secondary Endpoint

The secondary endpoint is included to assess the effects of treatment on tinnitus symptoms of participants based on the TFI score. Changes in symptoms of tinnitus as measured by TFI from the INTERIM visit to the FINAL visit will be reported.

[redacted]

15.6 Additional Endpoints

The following additional endpoints are included to assess the effects of treatment on quality of life as well as overall participant satisfaction with treatment:

Quality of Life/Satisfaction

(i) Changes in quality of life as measured by the HUI3 instrument from the SCREENING visit to the INTERIM visit and from the SCREENING visit to the FINAL visit will be reported.
(ii) Participant satisfaction rates with treatment as measured by the satisfaction questions at the FINAL visit will be reported.

[redacted]

[redacted]

All adverse events (AEs), and serious adverse events (SAEs), will be reported through treatment duration of 12 weeks. AEs and SAEs will be further categorised by the relationship to the investigational medical device (Adverse Device Effect, ADE) or an inadequacy of the investigational medical device (device deficiency). AEs will also be reported in relation to the severity of the AEs ('Mild', 'Moderate' or 'Severe' as defined in Section 16.3) as well as the specific onset and offset dates of the AEs. The onset/offset dates will be documented to characterize the transient or permanent nature of the AE's.

[redacted]

15.8 Missing Data

Every effort will be made to minimise the amount of missing data. Scheduled visits are expected and if not present, they will be considered as missing. Any missing data or data anomalies will be communicated to the site for clarification/resolution.

Participants who are missing THI score at the INTERIM visit or FINAL visit will be considered as missing data participants, for the analysis of the primary endpoint.

For the primary analysis of the primary endpoint, missing data will be handled as per the SAP. [redacted]

16.0 Safety Reporting

16.1 Definitions

Adverse events and device deficiencies shall be classified, evaluated and communicated to interested parties in accordance with ISO14155. Each AE shall be categorised as either serious adverse event or non-serious adverse event according to the definitions as provided in the ISO14155, and as detailed below. Each AE will have a causality determination assigned to it. AEs will be reported from the ENROLMENT visit to the FINAL visit.

Adverse Event (AE)

Any untoward medical occurrence, unintended disease or injury, or untoward clinical signs (including abnormal laboratory findings) in subjects, users or other persons, whether or not related to the investigational medical device and whether anticipated or unanticipated.

This definition includes events related to the investigational medical device or the comparator[1]; events related to the procedures involved. For users or other persons, this definition is restricted to events related to investigational medical devices.

Adverse Device Effect (ADE)

Adverse event related to the use of an investigational medical device.

This definition includes adverse events resulting from insufficient or inadequate instructions for use, deployment, implantation, installation, or operation, or any malfunction of the investigational medical device. This definition also includes any event resulting from use error or from intentional misuse of the investigational medical device. This definition also includes 'comparator' if the comparator is a medical device.

Device Deficiency

Inadequacy of a medical device with respect to its identity, quality, durability, reliability, usability, safety or performance.

Device deficiencies include malfunctions, use errors, and inadequacy in the information supplied by the manufacturer including labelling. This definition includes device deficiencies related to the investigational medical device or the comparator.

Serious Adverse Event (SAE)

Adverse event that led to any of the following:

a) death,
b) serious deterioration in the health of the subject, users, or other persons as defined by one or more of the following:
    1) a life-threatening illness or injury, or
    2) a permanent impairment of a body structure or a body function including chronic diseases, or
    3) in-patient or prolonged hospitalisation, or
    4) medical or surgical intervention to prevent life-threatening illness or injury or permanent impairment to a body structure or a body function,
c) foetal distress, foetal death, a congenital abnormality or birth defect including physical or mental impairment

Planned hospitalisation for a pre-existing condition, or a procedure required by the clinical investigation plan, without serious deterioration in health, is not considered a serious adverse event.

[1] It should be noted that the exact definitions following ISO14155 are listed here. The definitions include the use of comparator products. No comparators are used in this study and thus definitions are only applicable for the investigational medical device.

Serious Adverse Device Effect (SADE)

Adverse device effect that has resulted in any of the consequences characteristic of a serious adverse event.

Unanticipated Serious Adverse Device Effect (USADE)

Serious adverse device effect which by its nature, incidence, severity or outcome has not been identified in the current risk assessment.

Serious Health Threat

A serious health threat is a signal from any adverse event or device deficiency that indicates an imminent risk of death or a serious deterioration in the health in subjects, users or other persons, and that requires prompt remedial action for other subjects, users or other persons.

This would include events that are of significant and unexpected nature such that they become alarming as a potential serious health hazard or possibility of multiple deaths occurring at short intervals.

16.2 Causality

The ISO14155:2020 does not provide guidance on the causality assessment of adverse events the SAP will detail the definitions used to assess causality of non-serious adverse events. For the causality assessment of SAEs, the MDCG 2020-10/1 guideline will be followed.

For the purpose of harmonising reports, each SAE will be classified according to four different levels of causality:

- Not related
- Possible
- Probable
- Causal relationship

The sponsor and the investigators will use the following definitions to assess the relationship of the adverse event to the investigational device, the comparator or the investigation procedure.

Not related: relationship to the device, comparator or procedures can be excluded when:

- the event has no temporal relationship with the use of the investigational device or the procedures to application of the investigation device;
- the adverse event does not follow a known response pattern to the medical device (if the response pattern is previously known) and is biologically implausible;
- the discontinuation of medical device application or the reduction of the level of activation/exposure - when clinically feasible - and reintroduction of its use (or increase of the level of activation/exposure), do not impact on the adverse event;
- the event involves a body-site or an organ that cannot be affected;

- the adverse event can be attributed to another cause (e.g. an underlying or concurrent illness/ clinical condition, an effect of another device, drug, treatment or other risk factors);
- the event does not depend on a false result given by the investigational device used for diagnosis[2], when applicable;
- harms to the subject are not clearly due to use error;

In order to establish the relatedness, not all the criteria listed above might be met at the same time, depending on the type of device/procedures and the adverse event.

Possible: the relationship with the use of the investigational device or comparator, or the relationship with procedures, is weak but cannot be ruled out completely. Alternative causes are also possible (e.g. an underlying or concurrent illness/ clinical condition or/and an effect of another device, drug or treatment). Cases where relatedness cannot be assessed or no information has been obtained should also be classified as possible.

Probable: the relationship with the use of the investigational device or comparator, or the relationship with procedures, seems relevant and/or the event cannot be reasonably explained by another cause.

Causal relationship: the adverse event is associated with the investigational device, comparator, or with procedures beyond reasonable doubt when:

- the event is a known side effect of the product category the device belongs to or of similar devices and procedures;
- the event has a temporal relationship with investigational device use/application or procedures;
- the event involves a body-site or organ that
  - the investigational device or procedures are applied to;
  - the investigational device or procedures have an effect on;
- the adverse event follows a known response pattern to the medical device (if the response pattern is previously known);
- the discontinuation of medical device application (or reduction of the level of activation/exposure) and reintroduction of its use (or increase of the level of activation/exposure), impact on the adverse event (when clinically feasible);
- other possible causes (e.g., an underlying or concurrent illness/clinical condition and/or an effect of another device, drug or treatment) have been adequately ruled out;
- harm to the subject is due to error in use;
- the event depends on a false result given by the investigational device used for diagnosis[2], when applicable;

[2] If an investigational device gives an incorrect diagnosis, the patient might, for example, receive an unnecessary treatment and incur all the risks that accompany that treatment, or might be incorrectly diagnosed with a serious disease. In other cases, the patient might not receive an effective treatment (thereby missing out on the benefits that treatment would confer), or might not be diagnosed with the correct disease or condition.

CONFIDENTIAL: May not be reproduced, distributed or disclosed without prior consent from Neuromod Device Limited

In order to establish the relatedness, not all the criteria listed above might be met at the same time, depending on the type of device/procedures and the adverse event.

The Sponsor and the investigators will distinguish between the serious adverse events related to the investigational device and those related to the procedures (any procedure specific to the clinical investigation). An adverse event can be related both to procedures and the investigational device. Complications caused by concomitant treatments not imposed by the clinical investigation plan are considered not related. Similarly, several routine diagnostic or patient management procedures are applied to patients regardless of the clinical investigation plan. If routine procedures are not imposed by the clinical investigation plan, complications caused by them are also considered not related.

In some particular cases the event may be not adequately assessed because information is insufficient or contradictory and/or the data cannot be verified or supplemented. The Sponsor and the investigators will make the maximum effort to define and categorise the event and avoid these situations. Where an investigator assessment is not available and/or the sponsor remains uncertain about classifying the serious adverse event, the sponsor should not exclude the relatedness; the event should be classified as “possible” and the reporting not be delayed.

Particular attention shall be given to the causality evaluation of unanticipated serious adverse events. The occurrence of unanticipated events related to the use of the device could suggest that the clinical investigation places participants at increased risk of harm than was to be expected beforehand.

16.3 Severity

Each serious adverse event will be classified according to three (3) levels of severity. The Sponsor and the investigators will use the following definitions to assess the severity of the serious adverse event:

Mild: Awareness of signs or symptoms, but easily tolerated and are of minor irritant type causing no or minimal loss of time from normal activities. Symptoms do not require therapy or a medical evaluation; signs and symptoms are transient.

Moderate: Events introduce a low level of inconvenience or concern to the participant and may interfere with daily activities; moderate experiences may cause some interference with functioning.

Severe: Events that substantially interrupt the participant’s normal daily activities and generally require systemic drug therapy or other treatment; these events are usually incapacitating.

16.4 Reporting & Recording

Lenire is a CE-marked product to be used within its approved intended use. As such, vigilance requirements for CE-marked products will apply.

CONFIDENTIAL: May not be reproduced, distributed or disclosed without prior consent from Neuromod Device Limited

[redacted]

Any Device Deficiency shall be notified to the Sponsor and it should be documented in writing as to whether it could have led to a SADE.

All AEs shall be reported by the Investigator (or designee) to the Sponsor via the eCRF.

The sponsor shall implement and maintain a system to ensure that the reporting of SADEs will be provided by the investigator to the sponsor within 24 hours of learning of the adverse event.

The Investigator (or designee) shall provide source documents related to the adverse event as requested by the Sponsor.

SADEs must be sent directly to the Sponsor using the following email addresses and reported on the eCRF:

[redacted]

Furthermore, the Investigator (and/or Sponsor based on local/national requirements) shall report the event to the relevant competent authorities (CAs), ethics committees (ECs) and research offices in accordance with local/national requirements.

The Sponsor will evaluate all SADEs and send safety and vigilance reports to the relevant CAs, ECs and research offices in accordance with local/national requirements. Since the Medical Device Regulation is effective in where the investigation sites are located, timelines for reporting of SADEs to the CA as dictated by the MDR will be followed:

Safety reporting guidance under the MDR is provided in MDCG-2020-10/1. For Post Market Clinical Follow-Up investigations of CE-marked devices used within the intended use covered by the CE-marking, which is the case for the proposed investigation, reporting requirements of MDR Article 80(5) and (6) will apply. This means that the vigilance provisions laid down in Articles 87 to 90 (Vigilance and trend reporting and analysis of serious incidents, field safety corrective actions and vigilance data) and in the acts adopted pursuant to Article 91 shall apply for PMCF clinical investigations. This is laid down in Sponsors' SOPs (QM0025 Medical Device Reporting and Vigilance).

However reporting of serious adverse events where a causal relationship to the preceding investigational procedure has been established shall follow the reporting procedures of clinical investigations as outlined in Article 80, as described below.

The Sponsor must report to all the CAs where the clinical investigation is being conducted:

- For all reportable events (i.e., serious adverse events where a causal relationship to the preceding investigational procedure has been established) that indicate an

imminent risk of death, serious injury, or serious illness and that requires prompt remedial action for other participants/subjects, users or other persons or a new finding to it, Sponsor must report immediately, but not later **than 2 calendar days** after awareness by Sponsor of a new reportable event or of new information in relation with an already reported event. This includes events that are of significant and unexpected nature such that they become alarming as a potential public health hazard. It also includes the possibility of multiple deaths occurring at short intervals. These concerns may be identified by either the National Competent Authority or the manufacturer.

- Any other reportable events (i.e., serious adverse events where a causal relationship to the preceding investigational procedure has been established) or a new finding/update to it, Sponsor must report immediately, but not later **than 7 calendar days** following the date of awareness by the Sponsor of the new reportable event or of new information in relation with an already reported event.

All ADEs that result in a participant's withdrawal from the investigation or are present at the end of the investigation will be followed up until a satisfactory resolution occurs.

All unresolved SAEs will be followed up until the events are resolved, the participant is lost to follow-up, the participant has withdrawn consent, or the adverse event is otherwise explained.

Data collected from unresolved ADEs after the end of the investigation may be shared with the Sponsor, and subsequently with regulatory authorities, where applicable, to assess the benefit-risk profile of the device and for safety reporting requirements.

17.0 Investigation Management

The Sponsor and/or its delegated representatives will be responsible for data management, investigation design, ethics and regulatory approval, and investigation reporting requirements. The Sponsor and its delegated representatives will meet regularly as set out in the Project Plan. The Sponsor has delegated the activities that are listed in the signed work order relating to this investigation to the CRO. This is inclusive of any change orders requested throughout the investigation. All investigation specific activities have been delegated to the CRO, except specific device training required to configure and calibrate the device.

17.1 Inspection of Records

Investigators and Institutions will permit on site and remote monitoring and audits on behalf of the Sponsor and Regulatory Authorities. In the event of an audit or monitoring visit, the Investigators agree to allow the representatives of the Sponsor direct access to all investigation records and source documentation. In the event of Regulatory Inspection, the Investigators agree to allow inspectors direct access to all investigation records and source documentation.

Investigation data may be used as part of regulatory submissions and during regulatory audits.

17.2 Assessment and Management of Risk

As per section 17.3, 100% monitoring will be completed and as a result a risk assessment for a risk-based monitoring approach is not required. Monitors and auditors acting on behalf of the Sponsor will be allowed direct access to all site records and source documentation, either on site or remote access. Inspectors acting on behalf of Regulatory Authorities will be granted direct access to all site records and source documentation.

17.3 Monitoring

The Sponsor monitor or their delegated representative will conduct on site or remote visits to the investigation site prior to the start of the investigation and during the course of the investigation if required, in accordance with the Monitoring Plan. Monitoring will be performed according to ISO 14155 Clinical Investigation of Medical Devices for Human Subjects – Good Clinical Practice and where applicable, FDA guidelines. Following written SOPs, investigation monitors will verify that the investigation is conducted, and data are generated, documented and reported in compliance with the investigation plan, GCP and applicable regulatory requirements.

17.4 Early Investigation Termination

This is a post approval investigation of a CE marked investigational device and early termination is not anticipated. Adequate resources and planning have been put in place to ensure successful completion of the investigation; however, early termination may occur due to unforeseen circumstances. The Sponsor reserves the right to terminate the investigation at any time. In such cases, the Sponsor shall notify the relevant PIs and Ethics Committees of its decision.

Subsequently, the PIs will invite all participants back to the investigator site for an Early Discontinuation Visit and will request all participants to bring their devices. If participants are not willing to visit the investigator sites, the Sponsor will dispatch couriers to retrieve their devices in order to extract the compliance data. If participants refuse to return their devices, the Sponsor will endeavour to ensure the return of the device to extract compliance data. All participants will be treated as per standard of care at the investigation site after the investigation is terminated.

18.0 Good Clinical Practice and Ethical Considerations

18.1 Good Clinical Practice and Regulatory Requirements

This investigation will be performed in accordance with the following, as they apply to PMCF studies:

- Declaration of Helsinki
- ISO 14155:2020 Clinical Investigation of Medical Devices for Human Subjects – Good Clinical Practice
- MEDDEV 2.12/2 Rev 2 Post Market Clinical Follow-Up Studies
- Regulation (EU) 2016/679 (General Data Protection Regulation)
- Medical Devices Regulation (EU) 2017/745

CONFIDENTIAL: May not be reproduced, distributed or disclosed without prior consent from Neuromod Device Limited

- Pertinent individual country laws and regulations

18.2 Ethics Committee

The investigation shall not begin until the required approval/favourable opinion from the local/national EC and/or regulatory authority has been obtained, as appropriate.

Before participant recruitment, EC approval and/or any other required reviews of the investigation plan and relevant documentation by specific committees will be obtained in accordance with applicable local and country regulations. Any additional requirements imposed by the EC or regulatory authority shall be followed, if appropriate.

Annual EC approval and renewals will be obtained throughout the duration of the investigation as required by local/country or EC requirements. The Sponsor shall provide information on any reportable adverse events that may have occurred outside the control of the investigation in any country in which the device is legally marketed.

The Sponsor has the right at any time to terminate the investigation for clinical or administrative reasons or following advice from the PI. The end of the investigation will be reported to the EC as required by local/country or EC requirements.

18.3 Informed Consent Procedure

All consent materials will be developed by the investigator or designee if a local ethics board is used. Otherwise, consent forms will be developed by the Sponsor or designee and reviewed/customized by a central ethics board. Consent forms will be IRB/EC-approved and prepared in accordance with this clinical investigation plan, ISO 14155 and relevant regulatory requirements. The participant or their legally authorised representative will be asked to read and review the document prior to the start of the procedure.

All eligible candidates will receive a copy of the Participation Information Leaflet (PIL) and Informed Consent Form (ICF) and will have sufficient time to read and consider the information provided. These documents contain detailed information about participation, including the purpose of the investigation and what is required of them as investigation participants. The ICF will be available in the local language required by the member state and in line with the inclusion/exclusion criteria. Participants will have the opportunity to ask questions about the investigation, the PIL or ICF during the informed consent procedure. The patient will be asked to sign the informed consent form, acknowledging that they understand and desire to participate in the clinical investigation.

If they consent to participation, consent will be obtained by delegated site team members who will also provide their dated signature. Consent will be obtained at the SCREENING visit prior to any assessments being completed. A copy of the signed ICF, as well as any other written information, will be given to the participant. The original signed ICF will be retained at the investigation site. Participants will only be enrolled in this investigation if they meet all eligibility criteria and provide written informed consent. No investigation related data collection will occur prior to informed consent.

If new information regarding the investigational device becomes available and/or the clinical investigation plan changes and this information can significantly affect a participant's future health and medical care, participants will be informed of the information and may be asked to sign a revised informed consent form.

The *Subject Screening and Enrollment Log* will document all consented and enrolled participants and assign an investigation number. On all investigation-specific documents, other than the signed ICF, the participant will be referred to by this number, not by name. The *Subject Screening and Enrollment Log* and coded investigation-specific documents should be stored separate to each other.

18.4 Data Protection and Participant Confidentiality

All investigation personnel will comply with the requirements of GDPR and local data protection laws with regards to the collection, storage, processing and disclosure of personal information.

Investigation personnel outside of the direct care team will not access participant medical notes to assess eligibility unless previous consent was obtained to do so. Personnel outside of the direct care team will also not access participant medical notes to acquire participant contact details prior to receiving consent to do so, i.e., before participant has consented to investigation participation.

Investigation personnel will ensure that participant anonymity is maintained. Each participant will be assigned a unique identifier code (UIC) and will be identified only by this number on the CRFs and in any database, which will be password protected.

The *Subject Screening and Enrollment Log* will link personal participant information (name, date of birth and hospital/clinic number) to participant Investigation Number. This log should be stored separately to other investigation documentation and will only be accessible at the site.

The Sponsor will maintain a copy of all pseudonymised CRFs. Only the Sponsor team or their delegate will have access to these documents and investigation database off-site.

All documents will be stored securely and only accessible by authorised personnel. Medical information obtained by this investigation is confidential and may only be disclosed to third parties as permitted by the signed ICF, unless permitted or required by law. Where appropriate, medical information may be shared with a participant’s General Practitioner or other treating medical professions.

Documents and data generated by this investigation must be available for inspection upon request by Representatives of National and Local Health Authorities, the Sponsor, the EC, notified bodies, regulatory authorities (including FDA) or competent authorities, as appropriate.

18.5 Investigation Discontinuation and Closure

This investigation may be temporarily suspended or prematurely terminated if there is sufficient reasonable cause. Written notification, documenting the reason for investigation suspension or termination, will be provided by the Sponsor to investigators. Those investigation participants who consented to participate will be notified by their respective investigators, if required. If the investigation is prematurely terminated or suspended, the PI will promptly inform their IRB/EC and provide the reason(s) for the termination or suspension.

Circumstances that may warrant termination or suspension include, but are not limited to:

- Determination of unexpected, significant, or unacceptable risk to participants
- Unanticipated operational difficulties or other unforeseen occurrences

The investigation may resume if resolution of identified problems can be resolved.

18.6 Other Ethical Considerations

Severe cognitive impairment, assessed utilising the Mini Mental State Exam, is an exclusion criteria for this investigation. Participants must be willing and able to provide and understand informed consent.

18.7 Quality Assurance and Quality Control

Each site will be responsible for quality management of investigation conduct, data and if applicable biological specimen collection, analysis, documentation and completion in keeping with the ISO 14155, national, and local regulations.

Quality control (QC) procedures have been implemented with regard to the EDC (i.e., the database) system. Data QC checks that will be run on the database will be generated and documented in accordance with the CRO’s SOPs. Any missing data or data anomalies will be communicated to the site(s) for clarification/resolution. Sites should aim to resolve these queries as soon as possible in the EDC system.

The sites will provide direct access to all investigation related sites, source data/documents, and reports for the purpose of monitoring and auditing by the Sponsor, and inspection by local and regulatory authorities. All sites will follow their institutional procedures for collection and storage of medical records that will become part of this dataset.

[redacted]

[redacted]

[redacted]

CONFIDENTIAL: May not be reproduced, distributed or disclosed without prior consent from Neuromod Device Limited

[redacted]

21.0 References

1. J. Heller, Classification and epidemiology of tinnitus. Otolaryngol Clin North Am 36, 239-248 (2003).

2. McCormack, M. Edmondson-Jones, S. Somerset, D. Hall, A systematic review of the reporting of tinnitus prevalence and severity. Hear Res 337, 70-79 (2016).

3. D. Baguley, D. McFerran, D. Hall, Tinnitus. Lancet 382, 1600-1607 (2013).

4. Langguth B, Kreuzer PM, Kleinjung T, De Ridder D. Tinnitus: causes and clinical management. Lancet Neurol. 2013;12(9):920-930. doi:10.1016/S1474-4422(13)70160-1.

5. Hall DA, Láinez MJ, Newman CW, et al. Treatment options for participantive tinnitus: self-reports from a sample of general practitioners and ENT physicians within Europe and the USA. BMC Health Serv Res. 2011;11:302. Published 2011 Nov 4. doi:10.1186/1472-6963-11-302.

6. Rauschecker JP, Leaver AM, Mühlau M. Tuning out the noise: limbic-auditory interactions in tinnitus. Neuron. 2010;66(6):819-826. doi:10.1016/j.neuron.2010.04.032.

7. Møller, Aage R., et al., eds. Textbook of Tinnitus. Springer Science & Business Media, 2010.

8. Schaette R. Tinnitus in men, mice (as well as other rodents), and machines. Hear Res. 2014;311:63-71. doi:10.1016/j.heares.2013.12.004.

9. Roberts LE, Eggermont JJ, Caspary DM, Shore SE, Melcher JR, Kaltenbach JA. Ringing ears: the neuroscience of tinnitus. J Neurosci. 2010;30(45):14972-14979. doi:10.1523/JNEUROSCI.4028-10.2010.

10. Krauss P, Tziridis K, Metzner C, Schilling A, Hoppe U, Schulze H. Stochastic resonance-controlled upregulation of internal noise after hearing loss as a putative cause of tinnitus-related neuronal hyperactivity. Front Neurosci. 2016;10:597. Published 2016 Dec 27. doi:10.3389/fnins.2016.00597.

11. Ehret, Romand. The central auditory system. Oxford University Press. USA, 1997.

12. Yost, W. A. Fundamentals of hearing: an introduction. San Diego, CA: Academic. (2000).

13. Winer, Jeffery A. Decoding the auditory corticofugal systems. Hear Res. 207.1-2 (2005): 1-9.

14. Fritz JB, David SV, Radtke-Schuller S, Yin P, Shamma SA. Adaptive, behaviorally gated, persistent encoding of task-relevant auditory information in ferret frontal cortex. Nat Neurosci. 2010;13(8):1011-1019. doi:10.1038/nn.2598.

CONFIDENTIAL: May not be reproduced, distributed or disclosed without prior consent from Neuromod Device Limited

15. Rees, Adrian, and Alan R. Palmer, eds. The Oxford Handbook of Auditory Science: the Auditory Brain. Vol. 2. USA: Oxford university press, 2010.

16. Suga, Nobuo, et al. Corticofugal modulation and beyond for auditory signal processing and plasticity. Auditory and Vestibular Efferents. Springer, New York, NY, 2011. 313-352.

17. Markovitz CD, Hogan PS, Wesen KA, Lim HH. Pairing broadband noise with cortical stimulation induces extensive suppression of ascending sensory activity. J Neural Eng. 2015;12(2):026006. doi:10.1088/1741-2560/12/2/026006.

18. Winkowski, Daniel E., et al. Orbitofrontal cortex neurons respond to sound and activate primary auditory cortex neurons. Cereb Cortex. 28.3 (2017): 868-879.

19. Aitkin LM, Kenyon CE, Philpott P. The representation of the auditory and somatosensory systems in the external nucleus of the cat inferior colliculus. J Comp Neurol. 1981;196(1):25-40. doi:10.1002/cne.901960104.

20. Young ED, Nelken I, Conley RA. Somatosensory effects on neurons in dorsal cochlear nucleus. J Neurophysiol. 1995;73(2):743-765. doi:10.1152/jn.1995.73.2.743.

21. Levine RA. Somatic (craniocervical) tinnitus and the dorsal cochlear nucleus hypothesis. Am J Otolaryngol. 1999;20(6):351-362. doi:10.1016/s0196-0709(99)90074-1.

22. Marsh RA, Fuzessery ZM, Grose CD, Wenstrup JJ. Projection to the inferior colliculus from the basal nucleus of the amygdala. J Neurosci. 2002;22(23):10449-10460. doi:10.1523/JNEUROSCI.22-23-10449.2002.

23. Murray, Micah M., and Mark T. Wallace. The Neural Bases of Multisensory Processes. CRC Press, 2011.

24. Schofield BR, Motts SD, Mellott JG. Cholinergic cells of the pontomesencephalic tegmentum: connections with auditory structures from cochlear nucleus to cortex. Hear Res. 2011;279(1-2):85-95. doi:10.1016/j.heares.2010.12.019.

25. Basura GJ, Koehler SD, Shore SE. Multi-sensory integration in brainstem and auditory cortex. Brain Res. 2012;1485:95-107. doi:10.1016/j.brainres.2012.08.037.

26. Gruters KG, Groh JM. Sounds and beyond: multisensory and other non-auditory signals in the inferior colliculus. Front Neural Circuits. 2012;6:96. Published 2012 Dec 11. doi:10.3389/fncir.2012.00096.

27. Hormigo S, Horta Júnior Jde A, Gómez-Nieto R, López DE. The selective neurotoxin DSP-4 impairs the noradrenergic projections from the locus coeruleus to the inferior colliculus in rats. Front Neural Circuits. 2012;6:41. Published 2012 Jun 28. doi:10.3389/fncir.2012.00041.

CONFIDENTIAL: May not be reproduced, distributed or disclosed without prior consent from Neuromod Device Limited

28. Hurley LM, Sullivan MR. From behavioral context to receptors: serotonergic modulatory pathways in the IC. Front Neural Circuits. 2012;6:58. Published 2012 Sep 6. doi:10.3389/fncir.2012.00058.

29. De Ridder D, Elgoyhen AB, Romo R, Langguth B. Phantom percepts: tinnitus and pain as persisting aversive memory networks. Proc Natl Acad Sci U S A. 2011;108(20):8075-8080. doi:10.1073/pnas.1018466108.

30. De Ridder D, Vanneste S, Weisz N, et al. An integrative model of auditory phantom perception: tinnitus as a unified percept of interacting separable subnetworks. Neurosci Biobehav Rev. 2014;44:16-32. doi:10.1016/j.neubiorev.2013.03.021.

31. Tunkel DE, Bauer CA, Sun GH, et al. Clinical practice guideline: tinnitus. Otolaryngol Head Neck Surg. 2014;151(2 Suppl):S1-S40. doi:10.1177/0194599814545325.

32. Cima RFF, Mazurek B, Haider H, et al. A multidisciplinary European guideline for tinnitus: diagnostics, assessment, and treatment. Multidisziplinäre europäische Leitlinie für Tinnitus: Diagnostik, Einschätzung und Behandlung. HNO. 2019;67(Suppl 1):10-42. doi:10.1007/s00106-019-0633-7.

33. Tinnitus: assessment and management. London: National Institute for Health and Care Excellence (UK); March 11, 2020. https://www.nice.org.uk/guidance/ng155.

34. Bhatt JM, Lin HW, Bhattacharyya N. Prevalence, severity, exposures, and treatment patterns of tinnitus in the United States. JAMA Otolaryngol Head Neck Surg. 2016;142(10):959-965. doi:10.1001/jamaoto.2016.1700.

35. Ma X, Suga N. Augmentation of plasticity of the central auditory system by the basal forebrain and/or somatosensory cortex. J Neurophysiol. 2003;89(1):90-103. doi:10.1152/jn.00968.2001.

36. Weinberger NM. Associative representational plasticity in the auditory cortex: a synthesis of two disciplines. Learn Mem. 2007;14(1-2):1-16. Published 2007 Jan 3. doi:10.1101/lm.421807.

37. Engineer, Navzer D., et al. Reversing pathological neural activity using targeted plasticity. Nature. 470.7332 (2011): 101.

38. Koehler, Seth D., and Susan E. Shore. Stimulus-timing dependent multisensory plasticity in the guinea pig dorsal cochlear nucleus. PloS One. 8.3 (2013): e59828.

39. De Ridder, Dirk, et al. Safety and efficacy of vagus nerve stimulation paired with tones for the treatment of tinnitus: a case series. Neuromodulation. 17.2 (2014): 170-179.

40. Offutt, Sarah J., et al. Suppression and facilitation of auditory neurons through coordinated acoustic and midbrain stimulation: investigating a deep brain stimulator for tinnitus. J Neural Eng. 11.6 (2014): 066001.

CONFIDENTIAL: May not be reproduced, distributed or disclosed without prior consent from Neuromod Device Limited

41. Markovitz, Craig D., et al. Investigating a new neuromodulation treatment for brain disorders using synchronized activation of multimodal pathways. Sci Rep. 5 (2015): 9462.

42. Hamilton C, D'Arcy S, Pearlmutter BA, Crispino G, Lalor EC, Conlon BJ. An investigation of feasibility and safety of bi-modal stimulation for the treatment of tinnitus: an open-label pilot study. Neuromodulation. 2016;19(8):832-837. doi:10.1111/ner.12452.

43. Marks KL, Martel DT, Wu C, et al. Auditory-somatosensory bimodal stimulation desynchronizes brain circuitry to reduce tinnitus in guinea pigs and humans. Sci Transl Med. 2018;10(422):eaal3175. doi:10.1126/scitranslmed.aal3175.

44. Robards MJ. Somatic neurons in the brainstem and neocortex projecting to the external nucleus of the inferior colliculus: an anatomical study in the opossum. J Comp Neurol. 1979;184(3):547-565. doi:10.1002/cne.901840308.

45. Itoh K, Kamiya H, Mitani A, Yasui Y, Takada M, Mizuno N. Direct projections from the dorsal column nuclei and the spinal trigeminal nuclei to the cochlear nuclei in the cat. Brain Res. 1987 Jan 1;400(1):145-50. doi: 10.1016/0006-8993(87)90662-7. PMID: 2434184.

46. Ledoux JE, Ruggiero DA, Forest R, Stornetta R, Reis DJ. Topographic organization of convergent projections to the thalamus from the inferior colliculus and spinal cord in the rat. J Comp Neurol. 1987 Oct 1;264(1):123-46. doi: 10.1002/cne.902640110. PMID: 2445791.

47. Levine RA, Nam EC, Oron Y, Melcher JR. Evidence for a tinnitus subgroup responsive to somatosensory based treatment modalities. Prog Brain Res. 2007;166:195-207. doi: 10.1016/S0079-6123(07)66017-8. PMID: 17956783.

48. Vanneste S, Plazier M, Van de Heyning P, De Ridder D. Transcutaneous electrical nerve stimulation (TENS) of upper cervical nerve (C2) for the treatment of somatic tinnitus. Exp Brain Res. 2010;204(2):283-287. doi:10.1007/s00221-010-2304-5.

49. Gloeckner CD, Smith BT, Markovitz CD, Lim HH. A new concept for noninvasive tinnitus treatment utilizing multimodal pathways. Annu Int Conf IEEE Eng Med Biol Soc. 2013;2013:3122-3125. doi:10.1109/EMBC.2013.6610202.

50. Shore SE, Roberts LE, Langguth B. Maladaptive plasticity in tinnitus--triggers, mechanisms and treatment. Nat Rev Neurol. 2016;12(3):150-160. doi:10.1038/nrneurol.2016.12.

51. De Cicco V, Tramonti Fantozzi MP, Cataldo E, et al. Trigeminal, visceral and vestibular inputs may improve Cognitive Functions by Acting through the Locus Coeruleus and the Ascending Reticular Activating System: A New Hypothesis. Front Neuroanat. 2018;11:130. Published 2018 Jan 8. doi:10.3389/fnana.2017.00130.

CONFIDENTIAL: May not be reproduced, distributed or disclosed without prior consent from Neuromod Device Limited

52. Conlon B, Langguth B, Hamilton C, et al. Bimodal neuromodulation combining sound and tongue stimulation reduces tinnitus symptoms in a large randomized clinical study. Sci Transl Med. 2020;12(564):eabb2830. doi:10.1126/scitranslmed.abb2830.

53. Kleinstäuber, Maria, Ina Frank, and Cornelia Weise. "A confirmatory factor analytic validation of the Tinnitus Handicap Inventory." Journal of psychosomatic research 78.3 (2015): 277-284.

54. Landgrebe, Michael, et al. "Methodology of clinical trials for tinnitus." Textbook of tinnitus. Springer New York, 2011. 199-210

55. Zeman, Florian, et al. "Tinnitus Handicap Inventory for Evaluating Treatment Effects Which Changes Are Clinically Relevant?." Otolaryngology--Head and Neck Surgery 145.2 (2011): 282-287.

56. Horsman, J., Furlong, W., Feeny, D. and Torrance, G., 2003. The Health Utilities Index (HUI®): concepts, measurement properties and applications. Health and quality of life outcomes, 1(1), pp.1-13.

57. Spielberger CD, Gorsuch RL, Lushene PR, Vagg PR, Jacobs AG. 1983. Manual for the State-Trait Anxiety Inventory (Form Y). Consulting Psychologists Press, Inc.: Palo Alto

58. Folstein, M. F., S. E. Folstein, and P. R. McHugh. "Mini-mental state (MMSE) Journal of Psychiatric Research, 12." (1975): 189-198.

59. Monroe, Todd, and Michael Carter. "Using the Folstein Mini Mental State Exam (MMSE) to explore methodological issues in cognitive aging research." European Journal of Ageing 9.3 (2012): 265-274.

60. British Society of Audiology (2011) Pure-tone air- and bone-conduction threshold audiometry with and without masking. Reading: British Society of Audiology.

61. British Society of Audiology (2013) Recommended procedures for Tympanometry. Reading: British Society of Audiology

62. British Society of Audiology (2010) Ear Examination. Reading: British Society of Audiology.

63. Meikle MB, Henry JA, Griest SE, et al. The tinnitus functional index: development of a new clinical measure for chronic, intrusive tinnitus [published correction appears in Ear Hear. 2012 May;33(3):443]. Ear Hear. 2012;33(2):153-176. doi:10.1097/AUD.0b013e31822f67c0.

CONFIDENTIAL: May not be reproduced, distributed or disclosed without prior consent from Neuromod Device Limited

22.0 Appendices

Appendix 1 Schedule of Assessments

X = Mandatory, O = Optional/where applicable

	Screening	Treatment Phase				
VISIT	Visit 1 SCREENING Visit	Visit 2 ENROLMENT Visit	Compliance Call #1	Visit 3 INTERIM	Compliance Call #2	Visit 4 FINAL
TIMEFRAME	Week -10 (Max)	DAY 0	~Week 3	Week 6 (+/- 3W)	~Week 9	Week 12 (+/- 3W)
Informed Consent	X					
Medical History	X	O		O		O
Demographics	X					
Pregnancy Test	X					
Eligibility Assessment	X					
Tinnitus History	X					
THI	X	X		X		X
TFI	X	X		X		X
PTA	X	O		O		X
Otoscopy	X	O		O		O
Tympanometry	X	O		O		O
HUI Mark III	X			X		X
MMSE	X					
STAI	X					
Oral Assessment		X				X
Device Training/Fitting		X				
Device Compliance				X		X
Satisfaction Questionnaire						X
Compliance Call			X		X	
Case Report Forms (CRF)						
Visit 1 SCREENING CRF	X					

**Visit 2 ENROLMENT CRF**		X				
**Visit 3 INTERIM CRF**				X		
**Visit 4 FINAL CRF**						X
**Withdrawal CRF (where applicable)**		O		O		O
**Adverse Event CRF (where applicable)**		O	O	O	O	O
**Device Deficiciencies CRF (where applicable)**			O	O	O	O
**Concommitant Medications CRF (where applicable)**	O	O		O		O
**Concommitant Procedures**		O		O		O
**Investigation End CRF**	O	O	O	O	O	O
**Protocol Deviation CRF**	O	O	O	O	O	O
	Abbreviations: THI (Tinnitus Handicap Inventory), PTA (Pure Tone Audiometry), HUI (Health Utilities Index), MMSE (Mini-Mental State Examination), STAI (State-Trait Anxiety Inventory), TFI (Tinnitus Functional Index)					

[redacted]

[redacted]

CONFIDENTIAL: May not be reproduced, distributed or disclosed without prior consent from Neuromod Device Limited

[redacted]

CONFIDENTIAL: May not be reproduced, distributed or disclosed without prior consent from Neuromod Device Limited

[redacted]

CONFIDENTIAL: May not be reproduced, distributed or disclosed without prior consent from Neuromod Device Limited

[redacted]

[redacted]

CN0240 TENT-A3 Statistical Analysis Plan

for

TENT-A3

Neuromod Devices Limited

Treatment Evaluation of Neuromodulation for Tinnitus – Stage A3

CN0072

[redacted]

Author
[redacted]

[redacted]

[redacted]

[redacted]

TABLE OF CONTENTS

[redacted]

1 Abbreviations 8
2 Summary 9
3 Study Objectives and Endpoints 11
3.1 Study Objective 11
3.1.1 Primary Objective 11
3.1.2 Secondary Objectives 11
3.1.3 Additional objectives 11
3.2 Study Endpoints 11
3.2.1 Primary Endpoint 11
3.2.2 Secondary Endpoint 12
3.2.3 Additional Endpoints 12
4 Sample Size 12
5 Sequence of Planned Analyses 13
5.1 Interim Analyses 13
5.2 Final Analyses and Reporting 13
6 Analysis Populations 13
6.1 Intent-To-Treat Population (ITT) 13
6.2 Per-Protocol Population (PP) 13
7 General Issues for Statistical Analysis 14
7.1 Analysis Software 14
7.2 Disposition of Participants and Withdrawals 14
7.3 Methods for Withdrawals and Missing Data 15
[redacted]
7.7 Timing of Assessments and Events for Analysis 17
8 Demographics and Other Baseline Characteristics 18

8.1 Demographics 18
8.2 Prior and Concurrent Medications 18
8.3 Tinnitus History 18
8.4 Other Baseline Medical History 18
8.5 Measurement of Treatment Compliance 18
9 Effectiveness Analyses 19
9.1 Primary Variable 19
9.2 Secondary Variable 19
9.3 Additional Variables 20
10 Safety Analyses 20
11 Adverse Events 21
11.1 All Adverse Events 21
11.2 Adverse Events Leading to Withdrawal 21
11.3 Serious Adverse Events (SAE) 21
11.4 Procedure Related Adverse Events 22
11.5 Serious Procedure Related Adverse Events 22
11.6 Adverse Device Effect (ADE) 22
11.7 Serious Adverse Device Effect (SADE) 23
11.8 Unanticipated Serious Adverse Device Effect (USADE) 23
11.9 Device Deficiency 23
11.10 Deaths 23
12 Other Planned Analyses 23
12.1 Planned Subgroup Analyses 23
12.1.1 Gender 24
12.1.2 Tinnitus Severity 24

[redacted]

[redacted]

1 ABBREVIATIONS

ADE	Adverse Device Effect
AE	Adverse Event
CIP	Clinical Investigation Plan
CRF	Case Report Forms
CIR	Clinical Investigation Report
ETS	Electrical Tongue Stimulation
FCS	Fully Conditional Specification
FDA	United States Food and Drug Administration
HUI3	Health Utilities Index Mark III
ITT	Intent-To-Treat Population
MCMC	Markov Chain Monte Carlo
PP	Per-Protocol Population
PT	Preferred Term
PTA	Pure Tone Audiometry
SADE	Serious Adverse Device Effect
SAE	Serious Adverse Event
SAP	Statistical Analysis Plan
SOC	System Organ Category
STAI	State-Trait Anxiety Inventory
TENT-A3	Treatment Evaluation of Neuromodulation for Tinnitus Stage-A3
TFI	Tinnitus Functional Index
THI	Tinnitus Handicap Inventory
USADE	Unanticipated Serious Adverse Device Effect

[redacted]

2 SUMMARY

TITLE	Treatment Evaluation of Neuromodulation for Tinnitus – Stage A3
PREFACE	This Statistical Analysis Plan (SAP) describes the planned analysis and reporting for Neuromod Devices Limited protocol CN0072 (Treatment Evaluation of Neuromodulation for Tinnitus – Stage A3). This study continues to assess the safety and performance of the *Lenire®* device (CE 615889), and specifically assesses the clinical benefit of adding tongue stimulation to sound-only stimulation for the treatment of chronic subjective tinnitus in adult participants (at least 18 years of age), and whom have a minimum tinnitus severity and were experiencing tinnitus symptoms for a specified duration.<br><br>[redacted]
PURPOSE	The purpose of this SAP is to outline the planned analyses in support of the Clinical Investigation Report (CIR) for protocol CN0072. Exploratory analyses not necessarily identified in this SAP may be performed to support the clinical development programme. Any post-hoc, or unplanned analyses not identified in this SAP will be clearly identified in the respective CIR.
STUDY OBJECTIVES	**Primary**: the primary objective is to determine whether the addition of tongue stimulation to sound-only stimulation provides additional clinically significant improvement in tinnitus symptoms beyond that of the sound-only stimulation component as measured by the Tinnitus Handicap Inventory (THI).<br><br>**Secondary**: to determine the effect of treatment on the symptoms of tinnitus after the addition of tongue stimulation to sound-only stimulation as measured by the Tinnitus Functional Index (TFI).<br><br>**Additional**: to determine the effect of treatment on the quality of life of tinnitus sufferers as measured by the Health Utilities Index Mark III (HUI3) questionnaire and to assess treatment satisfaction based on two satisfaction questions.
STUDY DESIGN	This is a prospective, single arm, repeated measures, multi-site, post-market investigation of the *Lenire* CE marked medical device to continue to assess the safety and performance of two sequential 6-week stages of different stimulation settings of the investigational device in participants, aged over 18 years, with chronic subjective tinnitus. See CIP, Section “Investigation Design," for additional details.<br><br>Participants will be recruited through various channels as is standard practice at the investigation site and detailed in the relevant Recruitment Plan. If participants are deemed to be potentially eligible through an online pre-

	screening process, they will be invited to the clinic for a SCREENING visit (Visit 1). Before participants undergo any screening assessments for the investigation in the clinic, they will go through the informed consent process detailed in Section 18.3 “Informed Consent Procedure” of the CIP. If participants have provided written consent and are deemed eligible for enrolment at a SCREENING visit (Visit 1), they are invited to an ENROLMENT visit (Visit 2) where they are trained and fitted with the device. ENROLMENT will take place no more than 10 weeks after the initial SCREENING visit. The treatment consists of one device per participant and the treatment is intended to be self-administered in the participant’s home. Participants will be directed to complete two 30-minute treatment sessions each day throughout the 12-week period between the ENROLMENT and FINAL clinic visits.<br><br>The study schedule includes an INTERIM clinic visit (Visit 3) approximately 6 weeks after ENROLMENT. The parameter set is then updated from *PS6-No electrical tongue stimulation (ETS)* to *PS6* at this visit and compliance to treatment discussed with the participant. The FINAL visit (Visit 4) should be scheduled approximately 12 weeks after ENROLMENT. Unscheduled visits for withdrawal from the investigation or if required for adverse events can be scheduled at any time from ENROLMENT to the FINAL visit. The primary outcome measure, THI, is captured at every timepoint (including at withdrawal visits if possible).<br><br>Participants receive sound-only stimulation (*PS6 – No ETS*) during Stage 1 (ENROLMENT to INTERIM) and bimodal stimulation (*PS6*) during Stage 2 (INTERIM to FINAL).
**ENDPOINTS**	**Primary:** the primary endpoint is the responder rate in Stage 2 (the second 6-week period of treatment from INTERIM visit to FINAL visit comprising combined sound and tongue stimulation) compared to the point-estimate of the responder rate observed during Stage 1 (the first 6-week period of treatment from ENROLMENT visit to INTERIM visit comprising sound-only stimulation), where a responder is defined as a participant with an improvement in THI score of at least 7 points within the corresponding treatment stage.<br><br>**Secondary:** the secondary endpoint is to assess changes in symptoms of tinnitus as measured by TFI from the INTERIM visit to the FINAL visit.<br><br>**Additional:** the following additional endpoints are included to assess the effects of treatment on quality of life of participants as well as overall participant satisfaction with treatment:<br><br>Quality of Life/Satisfaction:

	(i) Changes in quality of life as measured by the HUI3 instrument from the SCREENING visit to the INTERIM visit and from the SCREENING visit to the FINAL visit will be reported.<br>(ii) Participant satisfaction rates with bimodal treatment as measured by the satisfaction questions at the FINAL visit will be reported.
**INTERIM ANALYSES**	No interim analyses are planned for this study.
**FINAL ANALYSES**	All final planned analyses identified in this SAP will be completed after all enrolled participants have completed their treatment or have been identified as 'lost to follow-up'.

3 Study Objectives and Endpoints

3.1 Study Objective

The objective of this clinical investigation is to determine whether the addition of tongue stimulation to sound-only stimulation provides additional clinically significant improvements in tinnitus symptoms beyond that of the sound-only stimulation component of the bimodal treatment.

3.1.1 Primary Objective

The primary objective is to determine whether the addition of tongue stimulation to sound-only stimulation provides additional clinically significant improvement in tinnitus symptoms beyond that of the sound-only stimulation component as measured by the THI.

3.1.2 Secondary Objectives

The secondary objective is to determine the effect of treatment on the symptoms of tinnitus after the addition of tongue stimulation to sound-only stimulation as measured by the TFI.

3.1.3 Additional objectives

The additional objectives are to determine the effect of treatment on the quality of life of tinnitus sufferers as measured by the HUI3 questionnaire and to assess treatment satisfaction based on two satisfaction questions.

3.2 Study Endpoints

3.2.1 Primary Endpoint

The primary endpoint is the responder rate in Stage 2 (the second 6-week period of treatment from INTERIM visit to FINAL visit comprising combined sound and tongue stimulation) compared to the point-estimate of the responder rate observed during Stage 1 (the first 6-week period of treatment from ENROLMENT visit to INTERIM visit comprising sound-only stimulation), where a responder is defined as a participant with an improvement in THI score of at least 7 points within the corresponding treatment stage.

3.2.2 Secondary Endpoint

The secondary endpoint is to assess changes in symptoms of tinnitus as measured by TFI in Stage 2 (the second 6-week period of treatment from INTERIM visit to FINAL visit comprising combined sound and tongue stimulation).

3.2.3 Additional Endpoints

The following additional endpoints are included to assess the effects of treatment on quality of life as well as overall participant satisfaction with treatment:

Quality of Life/Satisfaction

(i) Changes in quality of life as measured by the HUI3 instrument from the SCREENING visit to the INTERIM visit and from the SCREENING visit to the FINAL visit will be reported.
(ii) Participant satisfaction rates with bimodal treatment as measured by the satisfaction questions at the FINAL visit will be reported.

4 Sample Size

The investigation sample size calculation is based on the primary endpoint hypothesis that the responder rate in Stage 2 (the second 6-week period of treatment comprising combined sound and tongue stimulation) is greater than the point-estimate of the responder rate observed during Stage 1 (the first 6-week period of treatment comprising sound-only stimulation), where a responder is defined as a participant with an improvement in THI score of at least 7 points within the corresponding treatment stage.

The sample size calculation was performed with the following assumptions and specifications:

- Hypothesis testing will be performed using a single sample, one-sided normal approximation test (Z-test) for a binomial proportion.
- The null hypothesis is that the percentage of participants achieving at least 7 points reduction in THI score in Stage 2 (p_2) is less than or equal to the point-estimate of the responder rate in Stage 1 (p_1; H_0: $p_2 \leq p_1$).
- The alternative hypothesis is that the percentage of participants achieving at least 7 points reduction in THI score in Stage 2 (p_2) is greater than the point-estimate of the responder rate in Stage 1 (p_1; Ha: $p_2 > p_1$).
- An estimated responder rate of 45% for p_1 is based on relevant data from the previous TENT-A2 study and accounts for a reasonable upper bound for the placebo effect as observed in the literature.
- An estimated responder rate of 60% for p_2 is based on relevant data from the previous TENT-A2 study, where using modified Wald binomial probabilities with 90% confidence leads to an

estimated responder rate of at least 61% that is required and is then rounded to 60% to account for a worst-case scenario responder rate.

- Power (1- β) = 0.8
- Type I error rate (α) = 0.025

These specifications yielded a sample size estimate of 89 participants to complete all assessments in the clinical investigation.

The sample size was increased to 112 enrolled participants to allow for 20% dropouts or attrition during the study, including due to the current COVID-19 pandemic.

5 SEQUENCE OF PLANNED ANALYSES

5.1 INTERIM ANALYSES

There are no planned Interim Analyses for this study.

5.2 FINAL ANALYSES AND REPORTING

All final, planned analyses identified in the CIP and in this SAP will be performed only after all enrolled participants have completed their treatment or have been identified as 'lost to follow-up'. Key statistics and study results will be made available to the sponsor following database lock. Any post-hoc, exploratory analyses which were not identified in this SAP, will be documented, and reported as necessary. Any results from these unplanned analyses will also be clearly identified as post-hoc analyses.

6 ANALYSIS POPULATIONS

6.1 INTENT-TO-TREAT POPULATION (ITT)

The Intent-To-Treat (ITT) population will consist of all participants who meet the eligibility criteria, are enrolled in the investigation, and are fitted with the investigational device. 'Fitted' is defined as participants receiving a device configured for them, having completed a supervision session and are comfortable taking the device home with them.

This population will be utilised for the primary analysis of the primary endpoint and the analysis of the secondary and additional endpoints.

6.2 PER-PROTOCOL POPULATION (PP)

The Per-Protocol (PP) population will consist of all participants who

[redacted]

- meet the eligibility criteria as outlined in Section 12.2 “Inclusion Criteria” and Section 12.3 “Exclusion Criteria” of the CIP,
- are enrolled in the investigation,
- are fitted with the investigational device,
- and are compliant to minimum levels of device usage as outlined in Section 14.1 “Data Collection” of the CIP.

This analysis population will be utilised for the alternative analysis of the primary, secondary and additional endpoints.

7 General Issues for Statistical Analysis

The study will use a standard, frequentist approach to statistical analysis. Descriptive statistics (mean, standard deviation, frequencies, etc.) for baseline participant characteristics, participant disposition and other relevant study parameters will be reported.

Continuous variables will be summarised by the number of observations as well as mean, median, standard deviation, minimum, and maximum values. Categorical variables will be summarised using frequencies and percentages. Summaries will be reported for each study stage, as appropriate.

7.1 Analysis Software

Analysis data sets, statistical analyses and associated output generated by Avania will be generated using SAS® Software version 9.4 or later, and R version 4.1.2 or later.

7.2 Disposition of Participants and Withdrawals

Participants may stop using the device at any time or when instructed by the clinical provider to do so, e.g., if an adverse event occurs. Records relating to treatment will be kept, as this is valuable to the investigation.

Investigators can withdraw a participant if they become aware of any new information that contraindicates a participant for use of the device (e.g., becoming pregnant during the investigation).

The number and proportion of participants in each analysis population will be presented with percentages based on the ITT population.

All participants who provide written informed consent will be accounted for. The frequency and percent of participants who completed each scheduled assessment will be presented in a table.

The number and proportion of ITT participants prematurely withdrawing will be presented overall and by reason of discontinuation.

AVANIA
ADVANCING YOUR MEDICAL TECHNOLOGY

[redacted]

7.3 Methods for Withdrawals and Missing Data

Every effort will be made to minimise the amount of missing data. Scheduled visits are expected and if not present, after at least the minimum number of attempts to contact/reschedule the visit have failed (as per the CIP) or the participant does not return for their alternative scheduled visit, they will be considered as missing. Any missing data or data anomalies will be communicated to the site for clarification/resolution.

It is expected that THI questionnaires, necessary for the calculation of the primary endpoint, at the ENROLMENT, INTERIM or FINAL visit will be fully completed. THI questionnaires with any unanswered item at any visit will be considered missing questionnaires at that specific visit. The THI total score at the missing timepoint will then be imputed using the following variables in the imputation model:

- THI total score at all visits
- TFI total score at all visits
- Tinnitus duration (duration of initial tinnitus from onset at the SCREENING visit)
- Nature of the tinnitus at the SCREENING visit
- Prominent tinnitus sound at the SCREENING visit
- Tinnitus type at the SCREENING visit
- Age
- Gender
- Race
- Ethnicity

Data will be imputed in 2 steps:

1. All missing data for the predictors (except for THI total scores) listed above will be imputed 50 times in a single PROC MI in SAS software, using the MCMC method. 50 datasets with complete (non-missing) predictors will be created in this step.
2. Once missing data for the predictors have been imputed, missing THI total scores will then be imputed for each of the 50 imputed datasets created in Step 1, above. PROC MI will be employed for the imputation of THI scores at the SCREENING, ENROLMENT, INTERIM, and FINAL visits, using FCS linear regression method. Missing THI total scores will be imputed for each visit with separate FCS MODEL statements within a single PROC MI with nimpute=1, *using the 50 imputed datasets created in Step 1, and THI total score at all other visits*.

Participants are allowed to choose multiple race in the CRF. To avoid convergence issue of the imputation model, participants who select more than one option for race, will be grouped in the category “Multiple” for the purpose of the imputation. In the case that the imputation models fail to converge, variables will be removed one at a time in the reverse order that they are listed above until the model does converge. The complete THI total score at the ENROLMENT, INTERIM and the FINAL

visits will then be used to determine whether a participant achieved at least 7 points reduction in the THI score (0/1) at the end of Stage 1 and Stage 2. The primary endpoint hypothesis will then be tested for each one of the resulting 50 datasets, using a one sample, one-sided normal approximation test (Z-test) for a binomial proportion with a significance level of 0.025. The 50 Z-test results will then be combined using standard multiple imputation theory to obtain one overall p-value, which will be used to assess the primary endpoint after accounting for missing data.

[redacted]

[redacted]

7.7 Timing of Assessments and Events for Analysis

Study day 0 is the date of the ENROLMENT visit. Study days will be calculated as follows:

Study Day = Assessment/Event Date – Date of ENROLMENT visit

The following visits are planned for this study:

- Visit 1: SCREENING (Week -10 (max elapsed time))
- Visit 2: ENROLMENT (Week 0)
- Visit 3: INTERIM Visit (Week 6)
- Visit 4: FINAL Visit (Week 12)

Two treatment stages are planned for this study and are defined as:

Stage 1. ENROLMENT visit to INTERIM visit: sound-only stimulation (*PS6 – No ETS*)

Stage 2. INTERIM visit to FINAL visit: sound and tongue stimulation (*PS6*)

Treatment duration per participant is a maximum of 4 months, with a planned treatment duration of 12 weeks and an allowance of +/- 3 weeks for each treatment stage post enrolment to allow for scheduling difficulties. It's intended that participants will complete a minimum of 10 weeks of treatment.

Primary endpoint related data, THI questionnaire, will be assessed at the ENROLMENT, INTERIM and FINAL visits with additional data at SCREENING. Secondary endpoint related data, TFI questionnaire, will be assessed at the INTERIM and FINAL visits, as well as collecting additional data at SCREENING and ENROLMENT.

Additional endpoint related data, quality of life data, will be collected by the HUI3 instrument at the SCREENING, INTERIM and FINAL visits. Additional endpoint related data, participant satisfaction data, will be collected by the satisfaction questions at the FINAL visit.

All adverse events (AEs) will be reported through treatment duration of 12 weeks (Stage 1 and Stage 2).

8 Demographics and Other Baseline Characteristics

Measurements collected at the SCREENING visit will be considered as the baseline measurement. ITT population will be utilised for the analyses discussed in this section.

8.1 Demographics

Participant demographics and physical characteristics will be summarised by descriptive analysis. Gender, race, ethnicity, employment status, and military status will be summarised with frequency and percentage. Age will be summarised with mean, standard deviation, median, minimum, and maximum values.

8.2 Prior and Concurrent Medications

All prior and concomitant prescription medications collected in the CRFs, will be presented in a listing with medication name, route of administration, dose and other information located on the CRF.

8.3 Tinnitus History

Participant's tinnitus history such as location, type, sound description, nature of tinnitus, and whether General Practitioner or hearing specialist (e.g., audiologist or ENT) have been consulted will be summarised by frequency and percentage. Numerical summary of tinnitus duration (duration of initial tinnitus from onset at the SCREENING visit) will also be included.

8.4 Other Baseline Medical History

The CRF will record the participant's relevant medical history. Number and proportion of participants with each medical history listed in the CRF will be summarised in a table.

8.5 Measurement of Treatment Compliance

The *Lenire* device records the times and duration of use to an inbuilt memory chip. When the participant brings back their device, this usage data will be extracted and used for subsequent per-protocol analysis. This data will provide information regarding device usage and compliance with the treatment plan. Participants will be classified as either compliant or non-compliant to treatment plan, where the former is defined as at least 18 hours of device usage between ENROLMENT and INTERIM visits as well as at least 18 hours of device usage between INTERIM and FINAL visits.

Total duration of device usage and per treatment stage device usage will be summarised in a table numerically. Number and percent of compliant and non-compliant participants will also be provided for total treatment period and for each treatment stage.

[redacted]

9 Effectiveness Analyses

9.1 Primary Variable

The primary endpoint is the responder rate (percentage of participants achieving at least 7 points reduction in THI score) in Stage 2 of the study (from the INTERIM visit to the FINAL visit), attributed to the addition of tongue stimulation to sound-only stimulation, compared to the point-estimate of the responder rate in Stage 1 (from the ENROLMENT visit to INTERIM visit) for sound-only stimulation. The null and alternative statistical hypotheses for this endpoint are as follows:

$$H_0\colon \mathrm{p}_2 \leq \mathrm{p}_1$$

$$H_1\colon \mathrm{p}_2 > \mathrm{p}_1$$

Where $\mathbf{p_1}$ is the responder rate observed in Stage 1 (sound-only stimulation; the performance goal), and $\mathbf{p_2}$ is the responder rate in Stage 2 (addition of tongue stimulation to sound-only stimulation). Responder is defined as a participant with clinically meaningful reduction in THI score (of at least 7 points) within the corresponding treatment stage. The null hypothesis will be tested using a single sample, one-sided normal approximation test (Z-test) for a binomial proportion with a significance level of 0.025.

[redacted]

9.2 Secondary Variable

The secondary endpoint is included to assess the effects of treatment on tinnitus symptoms of participants based on the TFI score. Changes in symptoms of tinnitus as measured by TFI from the INTERIM visit to the FINAL visit will be reported.

[redacted]

[redacted]

[redacted]

9.3 Additional Variables

The following additional endpoints are included to assess the effects of treatment on quality of life as well as overall participant satisfaction with treatment:

Quality of Life/Satisfaction

(i) Changes in quality of life as measured by the HUI3 instrument from the SCREENING visit to the INTERIM visit and from the SCREENING visit to the FINAL visit will be reported.
(ii) Participant satisfaction rates with treatment as measured by the satisfaction questions at the FINAL visit will be reported.

[redacted]

10 Safety Analyses

All adverse events (AEs), and serious adverse events (SAEs), will be reported through treatment duration of 12 weeks. AEs and SAEs will be further categorised by the relationship to the investigational medical device (Adverse Device Effect, ADE) or an inadequacy of the investigational medical device (device deficiency). AEs will also be reported in relation to the severity of the AEs ('Mild', 'Moderate' or 'Severe') as defined in Section 16.3 "Severity" of the CIP, as well as the specific onset and offset dates of the AEs. The onset/offset dates will be documented to characterise the temporal nature of the AE's.

[redacted]

[redacted]

11 Adverse Events

Adverse events and device deficiencies shall be classified, evaluated, and communicated to interested parties in accordance with ISO14155. Each AE shall be categorised as either a serious adverse event or a non-serious adverse event according to the definitions as provided in ISO14155, and as detailed in Section 16.3 "Severity" of the CIP. Each AE will have a causality determination assigned to it. All AE analyses discussed in this section will be reported from the ENROLMENT visit to the FINAL visit in total and by treatment stage (ENROLMENT to INTERIM visit and INTERIM to FINAL visit) on the ITT population.

11.1 All Adverse Events

Summaries of incidence rates of specific AE types will be prepared. Because a participant may experience more than one AE, summaries will provide both the number of participants experiencing at least one event and the number of events from the ENROLMENT visit to the FINAL visit. Percentages provided will be the proportion of participants experiencing one or more adverse events. In addition, incidence of AEs will be presented by severity (mild, moderate, or severe) and by relationship to the investigational device and investigational procedures. Participants experiencing an event within a given Preferred Term (PT) and System Organ Class (SOC) more than once will be counted under the maximum severity experienced.

[redacted]

11.2 Adverse Events Leading to Withdrawal

A summary of incidence rates (frequencies and percentages) of AEs leading to study withdrawal by SOC (as applicable) will be prepared. A data listing of AEs leading to withdrawal will also be provided, displaying details of the events captured in the CRF.

11.3 Serious Adverse Events (SAE)

Serious adverse events are adverse events that led to any of the following:

a) death,

[redacted]

b) serious deterioration in the health of the subject, users, or other persons as defined by one or more of the following:
   1) a life-threatening illness or injury, or
   2) a permanent impairment of a body structure or a body function including chronic diseases, or
   3) in-patient or prolonged hospitalisation, or
   4) medical or surgical intervention to prevent life-threatening illness or injury or permanent impairment to a body structure or a body function, or

c) foetal distress, foetal death, a congenital abnormality, or birth defect including physical or mental impairment.

Planned hospitalisation for a pre-existing condition, or a procedure required by the clinical investigation plan, without serious deterioration in health, is not considered a serious adverse event.

Summaries of incidence rates and relationship to the investigational device/procedure of individual SAEs by SOC and PT (as applicable) will be prepared. Summaries will provide both the number of participants and the number of events. Percentages provided will be the proportion of participants experiencing one or more serious adverse events. A data listing of SAEs will also be provided, displaying details of the events captured on the CRF.

11.4 Procedure Related Adverse Events

Summaries of incidence rates of procedure related AEs by SOC and PT (as applicable) will be prepared. Summaries will provide both the number of participants and the number of events. Percentages provided will be the proportion of participants experiencing one or more procedure related AEs. Data listings of procedure related AEs will also be provided, displaying details of the events captured on the CRF.

11.5 Serious Procedure Related Adverse Events

Summaries of serious procedure related adverse events by SOC and PT (as applicable) will be prepared. Summaries will provide both the number of participants and the number of events. Percentages provided will be the proportion of participants experiencing one or more serious procedure related AEs. Data listings will also be provided, displaying details of the events captured on the CRF.

11.6 Adverse Device Effect (ADE)

Adverse device effect is an adverse event related to the use of an investigational medical device that is not a procedure related adverse event.

This definition includes adverse events resulting from insufficient or inadequate instructions for use, deployment, implantation, installation, or operation, or any malfunction of the investigational medical

device. This definition also includes any event resulting from use error or from intentional misuse of the investigational medical device.

Summaries of incidence rates of ADEs by SOC and PT (as applicable) will be prepared. Summaries will provide both the number of participants and the number of events. Percentages provided will be the percent of participants experiencing one or more ADE. Data listings of ADEs will also be provided, displaying details of the events captured on the CRF.

11.7 Serious Adverse Device Effect (SADE)

Serious adverse device effect is an adverse device effect that has resulted in any of the consequences characteristic of a serious adverse event.

Summaries of incidence rates of SADEs by SOC and PT (as applicable) will be prepared. Summaries will provide both the number of participants and the number of events. Percentages provided will be the proportion of participants experiencing one or more SADE. Data listings of SADEs will also be provided, displaying details of the events captured on the CRF.

11.8 Unanticipated Serious Adverse Device Effect (USADE)

Serious adverse device effect which by its nature, incidence, severity, or outcome has not been identified in the current risk assessment. If any USADE occurs, a data listing of USADEs will be provided, displaying details of the events captured on the CRF.

11.9 Device Deficiency

Device deficiency is an inadequacy of a medical device with respect to its identity, quality, durability, reliability, usability, safety, or performance.

A data listing of device deficiencies will be provided, displaying deficiency type, specification of device part, deficiency description, whether associated with an AE, and whether led or might have led to a SAE. Device deficiencies will be reported in total and also by treatment stage.

11.10 Deaths

Should any participants die during the TENT-A3 trial, relevant information will be supplied in a data listing.

12 Other Planned Analyses

12.1 Planned Subgroup Analyses

Subgroup analyses may be reported on the PP population and the ITT population (complete cases and with imputed missing values as outlined in **Section 7.3: Methods for Withdrawals and Missing Data)**.

[redacted]

12.1.1 Gender

Number of the responders and responder rates at the end of Stage 1 and Stage 2 for the primary endpoint will be reported in a table, including 95% confidence intervals for the responder rates, by gender, Male versus Female.

12.1.2 Tinnitus Severity

THI analyses relating to the primary endpoint will be reported based on THI severity categories assessed at ENROLMENT visit or INTERIM visit.

THI analyses relating to the primary endpoint will be reported on participants who meet the THI inclusion criterion (THI greater than or equal to 38) at ENROLMENT visit or INTERIM visit.

[redacted]

[redacted]

[redacted]

[redacted]
