# Supplementary material for: Combining sound with tongue stimulation for the treatment of tinnitus: a multi-site single-arm controlled pivotal trial
Source: Nat Commun. 2024 Aug 19;15:6806. doi: 10.1038/s41467-024-50473-z (PMC11333749; doi:10.1038/s41467-024-50473-z)
Supplement: Supplementary file 3 — Reporting Summary [file 41467_2024_50473_MOESM3_ESM.pdf]

nature portfolio

Corresponding author(s): Hubert H. Lim

Last updated by author(s): May 28, 2024

Reporting Summary

Nature Portfolio wishes to improve the reproducibility of the work that we publish. This form provides structure for consistency and transparency in reporting. For further information on Nature Portfolio policies, see our Editorial Policies and the Editorial Policy Checklist.

Statistics

For all statistical analyses, confirm that the following items are present in the figure legend, table legend, main text, or Methods section.

n/a	Confirmed	
☐	☒	The exact sample size (n) for each experimental group/condition, given as a discrete number and unit of measurement
☐	☒	A statement on whether measurements were taken from distinct samples or whether the same sample was measured repeatedly
☐	☒	The statistical test(s) used AND whether they are one- or two-sided<br>*Only common tests should be described solely by name; describe more complex techniques in the Methods section.*
☐	☒	A description of all covariates tested
☐	☒	A description of any assumptions or corrections, such as tests of normality and adjustment for multiple comparisons
☐	☒	A full description of the statistical parameters including central tendency (e.g. means) or other basic estimates (e.g. regression coefficient) AND variation (e.g. standard deviation) or associated estimates of uncertainty (e.g. confidence intervals)
☐	☒	For null hypothesis testing, the test statistic (e.g. F, t, r) with confidence intervals, effect sizes, degrees of freedom and P value noted<br>*Give P values as exact values whenever suitable.*
☐	☒	For Bayesian analysis, information on the choice of priors and Markov chain Monte Carlo settings
☒	☐	For hierarchical and complex designs, identification of the appropriate level for tests and full reporting of outcomes
☒	☐	Estimates of effect sizes (e.g. Cohen's d, Pearson's r), indicating how they were calculated

Our web collection on statistics for biologists contains articles on many of the points above.

Software and code

Policy information about availability of computer code

Data collection	Data were collected using a commercial clinical study database management system DFdiscover maintained by the study CRO, Avania.
Data analysis	Standard statistical methods in SAS® Software version 9.4 or later and R versions 4.1.2 or later were used by external CRO/Avania to perform the analyses, which were plotted/presented with Excel version 2403 or STATA version 15 in the manuscript.

For manuscripts utilizing custom algorithms or software that are central to the research but not yet described in published literature, software must be made available to editors and reviewers. We strongly encourage code deposition in a community repository (e.g. GitHub). See the Nature Portfolio guidelines for submitting code & software for further information.

Data

Policy information about availability of data

All manuscripts must include a data availability statement. This statement should provide the following information, where applicable:

- Accession codes, unique identifiers, or web links for publicly available datasets
- A description of any restrictions on data availability
- For clinical datasets or third party data, please ensure that the statement adheres to our policy

All relevant data associated with the published study are present in the paper or the Supplementary Information. Data related to the primary results as presented in the paper are available under restricted access as ethical approval is required as additional processing or analysis by third parties not involved in the clinical study was not covered in the approved protocol or patient consents. Access can be obtained, contingent on appropriate ethics approval and data sharing agreements, by

contacting HHL (tent-admin@tinnitustrial.ie) for the purposes of confirming the analysis in the paper. Responses to valid requests will be reasonably attempted and initiated within 10 working days of receipt beginning 3 months and ending 5 years after this article publication. The raw individual level participant data are not available due to data protection regulations in Europe and since the informed consent form signed by participants does not allow for sharing individual level participant data to third parties outside of the scope of the study.

Research involving human participants, their data, or biological material

Policy information about studies with human participants or human data. See also policy information about sex, gender (identity/presentation), and sexual orientation and race, ethnicity and racism.

Reporting on sex and gender	Primary endpoint analyses using Intention to Treat (ITT) for the full cohort and moderate or worse severity group were carried out according to participants' self-reported sex. Of the 112 enrolled participants, 77 were male and 35 were female.
Reporting on race, ethnicity, or other socially relevant groupings	Race, ethnicity and social groupings are not reported.
Population characteristics	Total number of participants at enrollment = 112. Mean age of the full enrolled cohort = 48.9 years. Mean Tinnitus Handicap Inventory (THI) score for the full enrolled cohort (n=112) at screening was 50.1 points.
Recruitment	Participants were recruited in 3 sites/clinics in Belgium, Germany and Ireland. Recruitment was achieved through advertising on online forums or via online and radio advertisements. Enrollment was on the basis of inclusion/exclusion criteria stipulated in the protocol. There were no potential self-selection bias or other biases present that the investigators are aware of.
Ethics oversight	The study was approved by the Research Ethics Committee of Universitair Ziekenhuis Antwerpen in Belgium (BUN B3002021000174), Research Ethics Committee of Medizinische Hochscule Hannover in Germany (10199_BO_S_2022), and the National Office for Research Ethics Committees in Ireland (22-NREC-MD-005).

Note that full information on the approval of the study protocol must also be provided in the manuscript.

Field-specific reporting

Please select the one below that is the best fit for your research. If you are not sure, read the appropriate sections before making your selection.

☒ Life sciences ☐ Behavioural & social sciences ☐ Ecological, evolutionary & environmental sciences

For a reference copy of the document with all sections, see nature.com/documents/nr-reporting-summary-flat.pdf

Life sciences study design

All studies must disclose on these points even when the disclosure is negative.

Sample size	The estimated responder rate of 45% for Stage 1 was based on relevant data from the previous TENT-A2 clinical trial and accounts for a reasonable upper bound for the placebo effect as observed in the literature. The estimated responder rate for Stage 2 was based on relevant data from the previous TENT-A2 clinical trial, and using modified Wald binomial probabilities with 90% confidence leads to a required estimated responder rate of at least 61%, which was then rounded to 60% to account for a worst-case scenario responder rate. The power for sample size was (1-β) equal to 0.8 with a type 1 error rate (α) equal to 0.025. These specifications yielded a sample size estimate of 89 participants for the study. The sample size was increased to 112 to consider a 20% drop-out or attrition during the clinical investigation (i.e., 80% of 112 equals 89.6), including to accommodate the COVID-19 pandemic during the study. For the analysis of the cohort with a THI severity greater than or equal to 38 at the interim visit, and considering the conservative rule of thumb of np and n(1-p) greater than 10 at each treatment stage, 44 subjects in the cohort would be a large enough sample size to assume an approximately normal distribution for the difference of proportions; hence, justifying using a z-test for the hypothesis test, where p corresponds to the responder rate.
Data exclusions	Data for all 112 enrolled participants were included in the study.
Replication	Replication of the results from the clinical trial results were confirmed with real-world evidence and previously published studies.
Randomization	No randomization was possible because the study design was a single arm repeated measures design where each participant served as their own control, and with a criterion in which participants had to achieve a clinically significant improvement in tinnitus symptoms with bimodal treatment during Stage 2 above and beyond what was already achieved with sound therapy during Stage 1.
Blinding	Blinding was not possible in the study given the single arm repeated measures design. A sham controlled study was not possible because both sound and tongue components involve suprathreshold stimuli that participants are expecting during treatment and thus participants would know if they received a sham condition and would not be sufficiently blinded.

Reporting for specific materials, systems and methods

We require information from authors about some types of materials, experimental systems and methods used in many studies. Here, indicate whether each material, system or method listed is relevant to your study. If you are not sure if a list item applies to your research, read the appropriate section before selecting a response.

Materials & experimental systems

n/a	Involved in the study
☒	☐ Antibodies
☒	☐ Eukaryotic cell lines
☒	☐ Palaeontology and archaeology
☒	☐ Animals and other organisms
☐	☒ Clinical data
☒	☐ Dual use research of concern
☒	☐ Plants

Methods

n/a	Involved in the study
☒	☐ ChIP-seq
☒	☐ Flow cytometry
☒	☐ MRI-based neuroimaging

Clinical data

Policy information about clinical studies

All manuscripts should comply with the ICMJE guidelines for publication of clinical research and a completed CONSORT checklist must be included with all submissions.

Clinical trial registration	NCT05227365
Study protocol	The study protocol and statistical analyses plan are provided in the supplementary information.
Data collection	A single arm repeated measures study where each participant served as their own control was conducted at three sites; (BRAI3N, Belgium, PI: Michael Boedts; German Hearing Center Hannover, Germany, PI: Andreas Buechner and Thomas Lenarz; St. James's Hospital, Ireland, PI: Guan Khoo). A total of 112 participants enrolled between 21st March 2022 and 7th June 2022 completed 6 weeks of sound-only stimulation, followed by 6 weeks of bimodal stimulation.
Outcomes	The primary endpoint is the responder rate in Stage 2 (the second 6-week period of treatment comprising bimodal sound and tongue stimulation) compared to the point-estimate of the responder rate observed during Stage 1 (the first 6-week period of treatment comprising sound-only stimulation), where a responder is defined as a participant with an improvement in Tinnitus Handicap Inventory (THI) score of at least 7 points within the corresponding treatment stage. Additional secondary analyses based on the Tinnitus Functional Index (TFI) and exploratory analyses based on the Health Utilities Index Mark III (HUI3), as well as two satisfaction questions and safety outcomes are also presented in this paper.

Plants

Seed stocks	*Report on the source of all seed stocks or other plant material used. If applicable, state the seed stock centre and catalogue number. If plant specimens were collected from the field, describe the collection location, date and sampling procedures.*
Novel plant genotypes	*Describe the methods by which all novel plant genotypes were produced. This includes those generated by transgenic approaches, gene editing, chemical/radiation-based mutagenesis and hybridization. For transgenic lines, describe the transformation method, the number of independent lines analyzed and the generation upon which experiments were performed. For gene-edited lines, describe the editor used, the endogenous sequence targeted for editing, the targeting guide RNA sequence (if applicable) and how the editor was applied.*
Authentication	*Describe any authentication procedures for each seed stock used or novel genotype generated. Describe any experiments used to assess the effect of a mutation and, where applicable, how potential secondary effects (e.g. second site T-DNA insertions, mosiacism, off-target gene editing) were examined.*
